# Neuropilin-1 Controls Endothelial Homeostasis by Regulating Mitochondrial Function and Iron-Dependent Oxidative Stress

**DOI:** 10.1016/j.isci.2018.12.005

**Published:** 2018-12-11

**Authors:** Theo Issitt, Emy Bosseboeuf, Natasha De Winter, Neil Dufton, Gaia Gestri, Valentina Senatore, Anissa Chikh, Anna M. Randi, Claudio Raimondi

**Affiliations:** 1 Vascular Sciences, Imperial Centre for Translational and Experimental Medicine, National Heart and Lung Institute, Imperial College London, London W12 0NN, UK; 2 Division of Biosciences, Department of Cell and Developmental Biology, University College London, Gower Street, London WC1E 6BT, UK; 3 UCL Institute of Ophthalmology, University College London, 11-43 Bath Street, London EC1V 9EL, UK; 4 Blizard Institute, Barts and the London School of Medicine and Dentistry, Queen Mary University of London, London E1 2AT, UK

**Keywords:** Cell Biology, Functional Aspects of Cell Biology, Molecular Biology

## Abstract

The transmembrane protein neuropilin-1 (NRP1) promotes vascular endothelial growth factor (VEGF) and extracellular matrix signaling in endothelial cells (ECs). Although it is established that NRP1 is essential for angiogenesis, little is known about its role in EC homeostasis. Here, we report that NRP1 promotes mitochondrial function in ECs by preventing iron accumulation and iron-induced oxidative stress through a VEGF-independent mechanism in non-angiogenic ECs. Furthermore, NRP1-deficient ECs have reduced growth and show the hallmarks of cellular senescence. We show that a subcellular pool of NRP1 localizes in mitochondria and interacts with the mitochondrial transporter ATP-binding cassette B8 (ABCB8). NRP1 loss reduces ABCB8 levels, resulting in iron accumulation, iron-induced mitochondrial superoxide production, and iron-dependent EC senescence. Treatment of NRP1-deficient ECs with the mitochondria-targeted antioxidant compound mitoTEMPO or with the iron chelator deferoxamine restores mitochondrial activity, inhibits superoxide production, and protects from cellular senescence. This finding identifies an unexpected role of NRP1 in EC homeostasis.

## Highlights

• A subcellular pool of NRP1 localizes in the mitochondria of endothelial cells (ECs)
• NRP1 regulates mitochondrial function via ABCB8 transporter
• NRP1 loss induces iron accumulation and iron-dependent oxidative stress in ECs
• NRP1 protects ECs from iron-dependent premature cellular senescence

## Graphical Abstract

**Figure:**
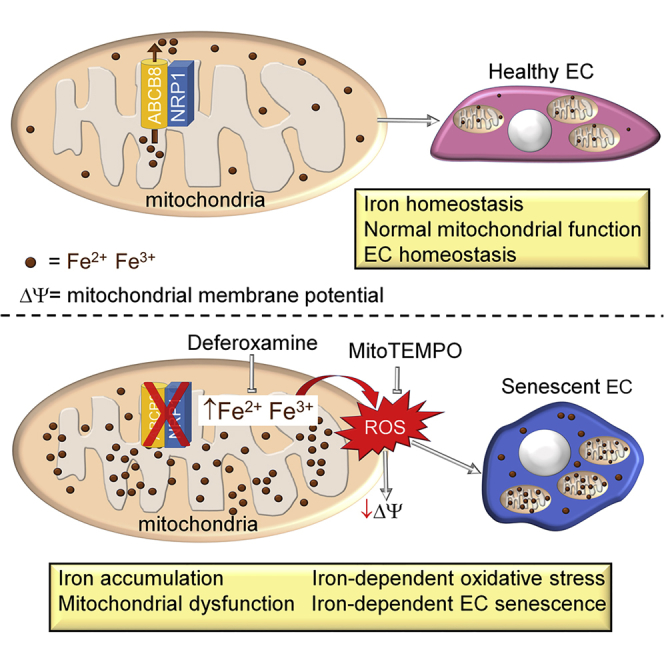

## Introduction

Neuropilin-1 (NRP1) is a transmembrane protein expressed by many cell types including endothelial cells (ECs) (Raimondi et al., 2016). Ablation of NRP1 expression in mice results in embryonic lethality caused by severe neural and vascular defects (Kawasaki et al., 1999, Kitsukawa et al., 1997). NRP1-dependent signaling pathways in ECs are essential for vascular development because endothelial-specific NRP1 knockout mice recapitulate the vascular defects of the global NPR1 knockout (Gu et al., 2003). ECs line the inner surface of blood vessels forming a quiescent selectively permeable monolayer and proliferate rarely over months or years (Augustin, 2004). In contrast, ECs proliferate and migrate in response to pro-angiogenic factors such as vascular endothelial growth factor (VEGF-A) during angiogenesis. NRP1 has been mainly investigated as a co-receptor for VEGF-A, but recent evidence shows that NRP1 also regulates VEGF-independent extracellular-matrix-mediated pathways that promote physiological and pathological angiogenesis (Fantin et al., 2015, Raimondi et al., 2014, Raimondi et al., 2015). Although the role of NRP1 in angiogenesis is well understood, the contribution of NRP1 signaling in the adult quiescent vasculature is less defined. The maintenance of endothelial homeostatic function over time is critical to prevent excessive permeability, thrombosis, and inflammation (Rajendran et al., 2013, Sena et al., 2013). In the adult vasculature, NRP1 has been implicated in VEGF-induced vascular permeability (Acevedo et al., 2008, Becker et al., 2005, Fantin et al., 2017, Raimondi et al., 2016, Roth et al., 2016) and in vascular leakage in a mouse model of diabetic retinal injury (Wang et al., 2015), but whether NRP1 has a broader role in EC homeostasis is poorly understood.

Mitochondria are emerging as regulators of EC signaling and function. ECs rely mainly on anaerobic glycolysis to meet their energy demand and to produce ATP (De Bock et al., 2013); thus mitochondrial contribution to EC energy production is limited in physiological conditions. However, mitochondria are essential for EC function because altering mitochondrial dynamics induces endothelial dysfunction (Shenouda et al., 2011). Accordingly, mitochondria regulate shear stress-induced vasodilation, hypoxia signaling, autophagy, and pro-inflammatory activation by producing mitochondrial reactive oxygen species (ROS), which act as a signaling molecule (Al-Mehdi et al., 2012, Liu et al., 2008). Conversely, fragmentation of the mitochondrial network occurs in ECs following exposure to oxidative stress (Jendrach et al., 2008), high glucose (Shenouda et al., 2011), and ischemia-reperfusion injury (Giedt et al., 2012, Makino et al., 2010). Although this evidence suggests that mitochondrial homeostasis and EC function are deeply entwined, the mechanism that regulates mitochondrial function in ECs is not completely understood.

Mitochondria are also a focal point for the regulation of cellular redox potential and iron metabolism. Mitochondria synthesize the iron-sulfur protein (Fe/S) clusters, which are essential for the electron transfer reactions and produce the heme's precursor compound protoporphyrin IX (Beinert et al., 1997, Hamza and Dailey, 2012, Lill et al., 1999). Thus mitochondria require iron to function and play a central role in iron metabolism. Defects in mitochondria biogenesis and morphology contribute to endothelial dysfunction and to the pathogenesis of cardiovascular diseases (Ong et al., 2010, Shenouda et al., 2011). Accordingly, the extent of atherosclerosis correlates with mitochondrial DNA damage in patients and in atherosclerosis mouse models (Ballinger et al., 2002). Furthermore, depletion of mitochondrial biogenesis regulator peroxisome proliferator-activated receptor γ coactivator 1α results in vascular dysfunction and inflammation because of increased mitochondrial ROS production in response to chronic angiotensin II infusion (Kroller-Schon et al., 2013).

ROS are mainly generated as a by-product of the mitochondria electron transport chain during oxidative phosphorylation (Turrens, 2003) or by accumulation of transition metals such as iron. Accumulation of ionic iron induces the formation of redox-active iron pools able to catalyze the production of free radical via Fenton chemistry (Kell, 2009, Silva-Gomes et al., 2014). ECs are equipped to withstand mechanical and chemical stresses and to maintain their function despite the physiological increases of cytokines, chemokines, and ROS during inflammation or infection. However, recent evidence shows that prolonged sustained ROS levels contribute to endothelial dysfunction and to the inflammatory response in atherosclerosis and coronary heart diseases (Cai and Harrison, 2000, Forstermann, 2008, Panth et al., 2016). Furthermore, elevated ROS levels induce premature EC senescence, which impairs EC function and homeostasis. In the aging cardiovasculature, senescent ECs accumulate in atherosclerotic lesions and likely contribute to disease progression by creating a pro-inflammatory and pro-thrombotic environment (Minamino et al., 2002, Vasile et al., 2001, Warboys et al., 2014).

Senescent cells irreversibly lose the ability to replicate and thereby contribute to tissue aging by disrupting tissue homeostasis (van Deursen, 2014). Replicative cell senescence is usually caused by progressive telomere shortening at each round of cell duplication (Cristofalo et al., 2004, Shay and Wright, 2005). In addition, DNA damage, oxidative stress, or mitochondrial dysfunction can cause premature senescence by telomere-independent mechanisms (Erusalimsky, 2009). EC senescence impairs vascular functions such as angiogenesis, nutrient trafficking, and vascular repair because it decreases proliferation and migration of ECs (Foreman and Tang, 2003).

Here, we found that in addition to the well-established role in angiogenesis, NRP1 also has a role in EC health in non-angiogenic conditions by promoting mitochondrial function through the mitochondria-specific ATP-binding cassette transporter ABCB8 (Ichikawa et al., 2012, Ichikawa et al., 2014). Downregulation of NRP1 increases iron levels in ECs and induces iron-dependent oxidative stress, which results in mitochondrial superoxide production, decreased mitochondrial activity, and EC senescence. As iron chelation restored mitochondrial activity, reduced mitochondrial superoxide, and reduced EC senescence in NRP1-deficient ECs, our findings indicate that NRP1 protects ECs from mitochondrial damage by reducing iron-dependent oxidative stress and thereby decreases premature EC senescence.

## Results

### NRP1 Localizes in the Mitochondria and Controls Mitochondrial Morphology in ECs Independently of VEGFR2

**Figure 1:**
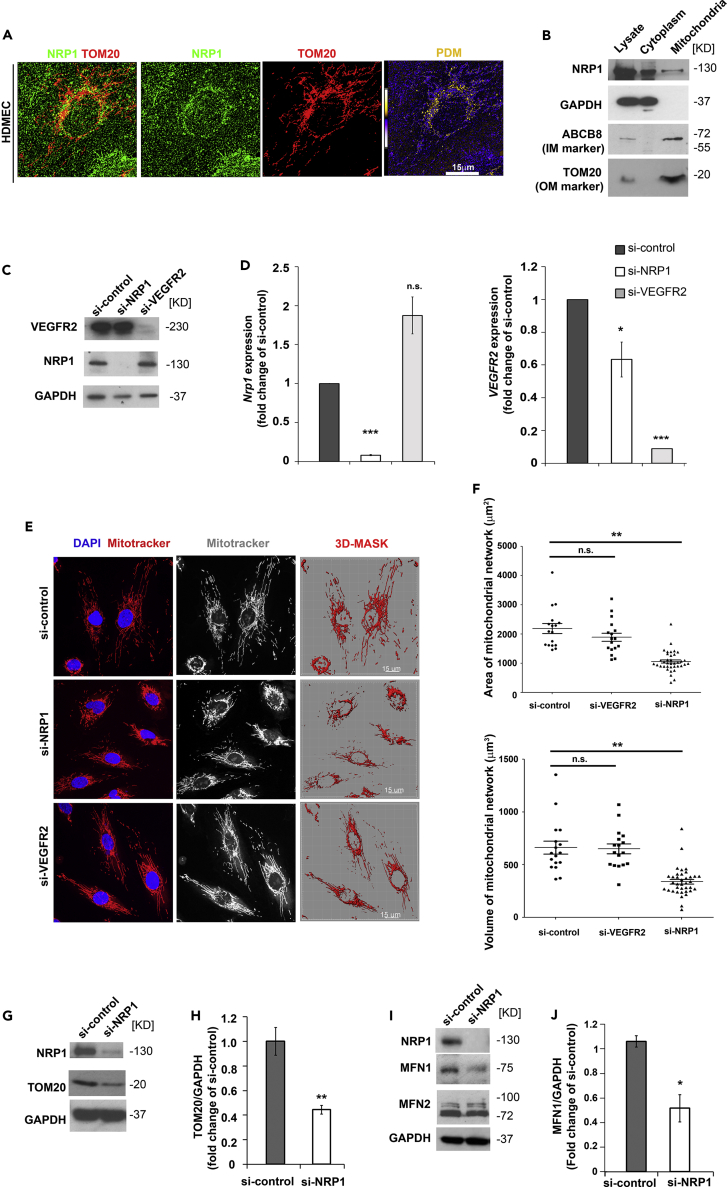
NRP1 Localizes in the Mitochondria and Regulates Mitochondrial Dynamics (A) HDMECs were stained for NRP1 (green) and TOM20 (red). Deconvoluted images of optical z stacks to visualize NRP1 and TOM20 co-localization; pseudo-colored “product of the differences from the mean” (PDM) images in which each pixel is equal to the PDM value are shown with a PDM scale; scale bar, 15 μm. (B) Mitochondrial and cytoplasmic fractions of HDMECs lysates were probed for NRP1, GAPDH, ABCB8, and TOM20. Immunoblotting representative of n = 4 experiments. (C) Representative immunoblot for NRP1 and VEGFR2 in HDMECs transfected with si-NRP1, si-VEGFR2, or si-control for 72 hr; n = 3. (D) Quantification of NRP1 and VEGFR2 mRNA by RT-qPCR in HDMECs transfected for 72 hr with si-NRP1, si-VEGFR2, or si-control. NRP1 and VEGFR2 levels were expressed as fold change of si-control (mean ± SEM; n = 3). (E) HDMECs transfected with the indicated si-RNAs were stained for mitochondria using MitoTracker (red) and counterstained with DAPI; scale bars, 15 μm. (F) Quantification of mitochondrial network in optical z stacks after applying a mask to quantify MitoTracker-positive areas and volumes (mean ± SEM; n = 3). (G) Immunoblotting for NRP1 and TOM20 in HDMECs transfected with si-NRP1 or si-control for 72 hr with GAPDH used as control. (H) TOM20 levels in immunoblots were quantified as pixel intensity relative to GAPDH, and values expressed as fold change of si-control (mean ± SEM; n = 3). (I) Immunoblotting for NRP1, MFN1, and MFN2 in HDMECs transfected with si-NRP1 or si-control for 72 hr with GAPDH used as control. (J) MFN1 levels in immunoblots were quantified as pixel intensity relative to GAPDH, and values expressed as fold change of si-control (mean ± SEM; n = 3). *p < 0.05, **p < 0.01, ***p < 0.001; n.s., not significant; Student's t test. See also Figure S1.

To investigate a potential role of NRP1 in mitochondria, we first investigated whether NRP1 localizes in the mitochondria of human microvascular endothelial cells (HDMECs) by performing immunostaining for NRP1 and the mitochondrial outer membrane component TOM20. Specificity of anti-NRP1 antibody was confirmed by staining HDMECs downregulated for NRP1 expression using a previously validated small interfering RNA (siRNA) (Fantin et al., 2015, Fantin et al., 2017, Raimondi et al., 2014) (Figures S1A and S1B). As expected, NRP1 was distributed throughout the cytoplasm and was enriched at the plasma membrane and in filopodia (Figure S1A). Co-localization analysis of de-convoluted high-magnification z stack, which generated pseudo-colored “product of the differences from the mean” (PDM) images in which each pixel is equal to the PDM value, showed that NRP1 co-localizes with TOM20 in the mitochondria (Figures 1A, S1C, S1C′, and S1D). We further investigated whether NRP1 is in close proximity with TOM20 by performing proximity ligation assay (PLA), which generates a fluorescent signal when target proteins are within a distance of 40 nm. Interestingly, HDMEC si-control showed a positive PLA signal, which was significantly decreased in HDMEC si-NRP1 (Figures S1E and S1F), suggesting that NRP1 localizes in the mitochondria. To confirm this finding, we performed mitochondrial fractionation. Cytoplasmic and mitochondrial fractions of HDMECs were immunoblotted for the cytoplasmic marker GAPDH and for the mitochondrial inner and outer membranes markers ABCB8 and TOM20, respectively (Figure 1B). Furthermore, fractions were immunoblotted for the endoplasmic reticulum (ER) marker KDEL and for the endosome marker EEA1 (Figure S1G). Importantly, the mitochondrial fraction was negative for cytoplasmic, endosomal, and ER markers, indicating that the mitochondrial fractions isolated were pure. Strikingly, NRP1 was detected by immunoblotting in the mitochondrial fraction (Figures 1B and S1G) as well as in the cytoplasmic fraction and in the whole lysate. Together these data show that a subcellular pool of NRP1 localizes into the mitochondria.

To determine whether NRP1 or VEGF signaling plays a role in mitochondrial function, we downregulated NRP1 or VEGFR2 expression in HDMECs and investigated mitochondrial morphology. Thus we analyzed the mitochondrial network coverage in HDMECs transfected with NRP1 targeting, VEGFR2-targeting, or with control siRNA (Figures 1C and 1D). Mitochondria were live stained with MitoTracker, and mitochondrial network area and volume were quantified by generating a 3D-rendered surface built on the MitoTracker fluorescence from optical z stacks acquired through high-resolution confocal microscopy (Figure 1E). Quantification revealed that NRP1-deficient, but not VEGFR2-deficient, HDMECs have reduced mitochondrial area and volume compared with control cells, suggesting that NRP1 regulates the extent of mitochondrial network independently of VEGF signaling (Figure 1F).

Then, we analyzed the protein level of the mitochondrial mass indicator TOM20 by immunoblotting, as a measurement of mitochondrial content (Burbulla et al., 2014). In agreement with the reduced mitochondrial network coverage (Figures 1E and 1F), NRP1 downregulation in HDMECs significantly reduced TOM20 levels compared with control cells, suggesting a decrease in mitochondrial mass in NRP1-deficient HDMECs (Figures 1G and 1H).

Because mitochondria are organized in a network maintained by organelle fission and fusion (Chan, 2006), we investigated whether NRP1 downregulation affects the expression of mitofusin-1 (MFN1) and mitofusin-2 (MFN2), which are known to promote mitochondrial fusion (Lugus et al., 2011, Ong et al., 2010). In agreement with the reduced mitochondrial network observed above (Figures 1E and 1F), we found that NRP1 downregulation significantly decreases MFN1, but not MFN2 expression (Figures 1I and 1J). Together, these data show that a pool of NRP1 localizes in the mitochondria and that NRP1 regulates mitochondrial mass and the organization of the mitochondrial network in ECs.

### NRP1 Protects Mitochondrial Activity and Prevents Oxidative Stress in ECs

**Figure 2:**
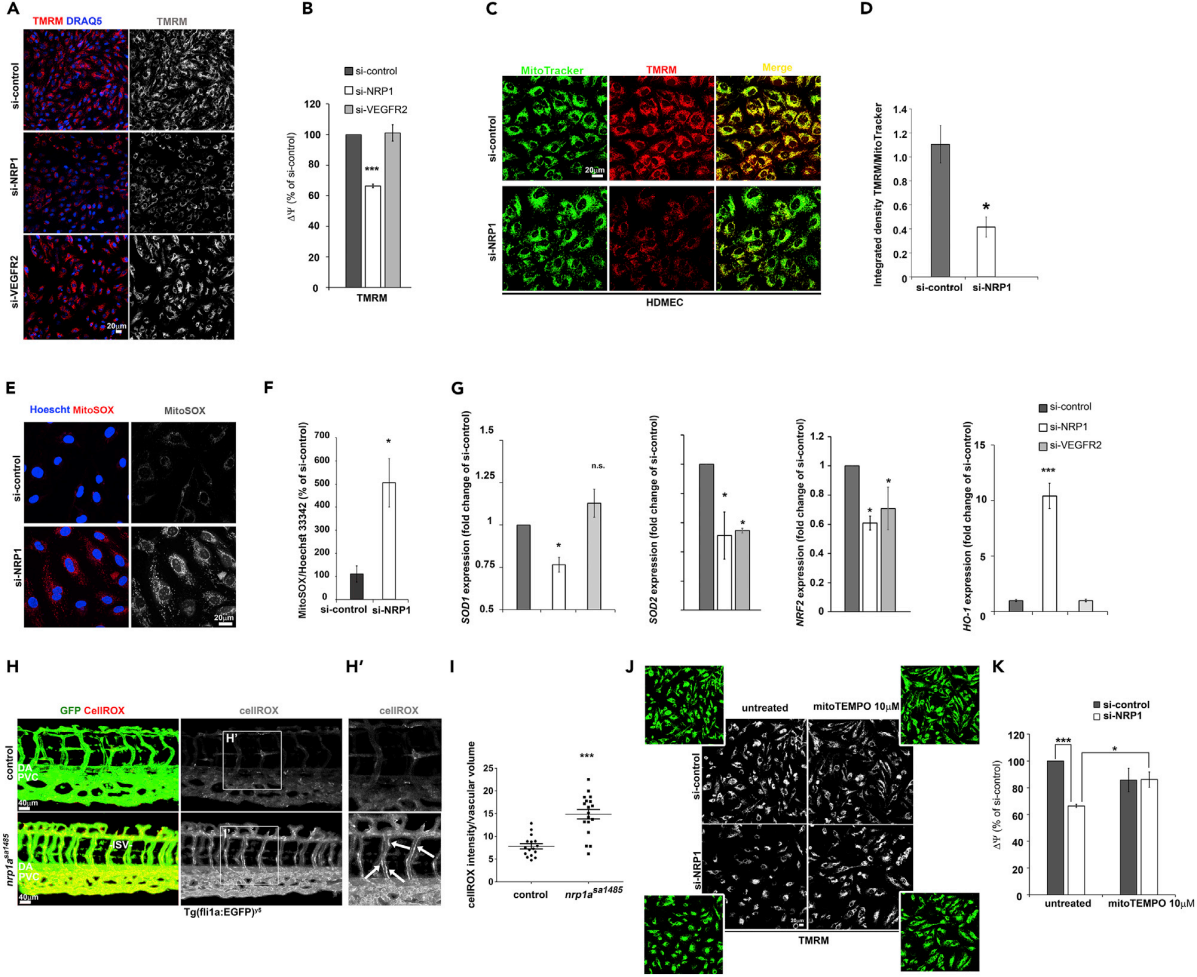
NRP1 Regulates Mitochondrial Activity and Suppresses Oxidative Stress (A) HDMECs transfected with si-NRP1, si-VEGFR2, or si-control were incubated with 100 nM TMRM and DRAQ5 (blue) and live imaged with a confocal microscope; scale bar, 20 μm. (B) Integrated density of TMRM signal per cell was quantified and expressed as percentage (mean ± SEM; n = 4) of si-control. (C) HDEMCs transfected with siNRP1 or si-control were incubated with 100 nM TMRM and 300 nM MitoTracker Deep Red FM; scale bar, 20 μm. (D) TMRM and MitoTracker Deep Red FM integrated density was calculated, and the TMRM/MitoTracker ratio visualized in the graph (mean ± SEM; n = 4). (E) HDMECs transfected with si-NRP1 or si-control were incubated with 5 μM MitoSOX (red and gray) and counterstained with Hoescht 33342 (blue); scale bar, 20 μm. (F) Integrated density of the MitoSOX signal per cell was quantified and expressed as percentage relative to si-control (mean ± SEM; n = 3). (G) Quantification of superoxide dismutase-1 and 2 (SOD1, SOD2), NRF2, and heme oxygenase-1 (HO-1) mRNA by RT-qPCR in HDMECs si-NRP1 or si-control expressed as fold change of si-control (mean ± SEM; n = 3). (H) *Tg*(*fli1a*:*egfp*)^*y5*^ wild-type and *nrp1a*^sa1485^ zebrafish embryos at 3 dpf were incubated with 2.5 μM CellROX (gray) in 10% DMSO for 45 min before imaging; scale bars, 40 μm. (H′) Higher-magnification images of area indicated in the square box in H; white arrows indicate ECs in ISV with high CellROX staining. (I) CellROX mean intensity was quantified in optical z stacks in n ≥ 16 embryos from three independent experiments after applying a mask to quantify CellROX-positive areas and volumes (mean ± SEM) within the vasculature. (J) HDMECs treated with mitoTEMPO 10 μM for 24 hr after 48 hr from transfection with si-NRP1 or si-control were incubated with 100 nM TMRM (gray); scale bar, 20 μm. (K) Integrated density of the TMRM signal per cell was quantified and normalized to cell number determined by manual counting using high-contrast images (panel J, green images) and expressed as percentage relative to si-control (mean ± SEM; n = 3). *p < 0.05, ***p < 0.001; Student's t test. See also Figure S2).

Because NRP1 loss reduces mitochondrial mass and mitochondrial network coverage, we investigated whether NRP1 promotes mitochondrial activity. In aerobic conditions, NADH and FADH2 produced during glycolysis, β-oxidation, and other catabolic processes are oxidized using molecular oxygen by the electron transport chain complexes. The transfer of electrons through the mitochondrial complexes I–IV located in the inner mitochondrial membrane provides the energy to generate an H^+^ gradient across the inner mitochondrial membrane, which creates an inter-membrane mitochondrial potential (ΔΨ) functional to ATP production. Thus we measured ΔΨ by live staining HDMECs transfected with si-NRP1, si-VEGFR2, or si-control with the fluorescent dye tetramethylrhodamine methyl ester (TMRM), whose accumulation into the mitochondria depends on ΔΨ (Scaduto and Grotyohann, 1999). Accordingly, treatment of HDMECs with the electron transport chain uncoupler carbonyl cyanide *m*-chlorophenylhydrazone completely abrogated TMRM mitochondrial accumulation (Figure S2A). Confocal analysis showed that NRP1 downregulation, but not VEGFR2 knockdown, significantly reduced TMRM staining compared with si-control (Figures 2A and 2B). Together, these data demonstrate that NRP1 regulates mitochondrial membrane potential and suggest that NRP1 promotes mitochondrial function in a VEGF-independent manner.

As NRP1 downregulation reduced the area and volume of the mitochondrial network (Figures 1E and 1F), we investigated whether the decreased TMRM staining observed in ECs lacking NRP1 was due to a reduced mitochondrial content or to a genuine reduction in the proton motive force. Thus we co-labeled HDEMCs and human umbilical vein endothelial cells (HUVECs) with TMRM and MitoTracker to measure simultaneously ΔΨ and mitochondrial content. Strikingly, NRP1 downregulation reduced the TMRM/MitoTracker ratio in HDMECs (Figures 2C and 2D) and HUVECs (Figures S2B and S2C). Furthermore, downregulation of NRP1 in HDMECs with two different NRP1-targeted siRNAs (Figure S2D) confirmed a reduction of TMRM/MitoTracker staining in cells lacking NRP1 expression (Figures S2E and S2F), indicating that the mitochondrial phenotype observed is not due to siRNA off-target effects. Together, these data indicate that lack of NRP1 expression in EC decreases mitochondrial proton motive force independently of the reduction in mitochondrial content.

As oxidative damage reduces mitochondrial activity and disrupts the organization of the mitochondrial network (Jendrach et al., 2008), we investigated whether NRP1 downregulation induces mitochondrial ROS production. HDMECs expressing or lacking NRP1 were live stained with the mitochondria-specific dye MitoSOX, which becomes fluorescent upon superoxide-mediated oxidation (Figure 2E). Strikingly, HDMECs lacking NRP1 expression showed significantly higher mitochondrial ROS compared with control, indicating that NRP1 expression suppresses mitochondrial ROS production (Figures 2E and 2F). Because inhibition of the endogenous antioxidant cellular system could lead to increased ROS levels, we investigated the role of NRP1 and VEGF signaling in the expression of the antioxidant enzymes superoxide dismutase 1 (SOD1), SOD2, and NRF2 in HDMECs. Interestingly, NRP1 downregulation, but not VEGFR2 knockdown, significantly reduced SOD1 expression compared with control cells (Figure 2G), whereas SOD2 and NRF2 expression was significantly reduced in cells transfected with either NRP1 or VEGFR2 siRNA (Figure 2G). Then, we investigated the expression of heme oxygenase 1 (HO-1), which is known to protect against oxidative stress and whose expression is strongly induced by a wide array of pro-oxidant and inflammatory stimuli (Luo et al., 2017). Downregulation of NRP1 induced a 10-fold upregulation of HO-1 expression compared with si-control, whereas VEGFR2 knockdown had no effect on HO-1 expression (Figure 2G). Accordingly, HO-1 protein level was significantly upregulated in HDMECs si-NRP1 (Figures S2G and S2H). Taken together, these data suggest that NRP1- and VEGF-mediated signaling pathways cooperate to maintain the cellular antioxidant defense system in HDMECs and that NRP1 loss induces an oxidative stress response that leads to HO-1 upregulation in ECs.

To further study the role of NRP1 in oxidative stress, we investigated whether NRP1 loss induces oxidative stress *in vivo* by measuring oxidative stress in the Tg(fli1a:EGFP)^y5^-labeled vasculature of *nrp1a*^*sa1485/sa1485*^ mutant (*nrp1a*^−/-^) zebrafish embryos. Live transgenic embryos at 3 days post-fertilization (dpf) were stained with CellROX dye, which detects generalized oxidative stress (Mugoni et al., 2014). To measure ROS specifically in the vasculature, we quantified CellROX fluorescence in isolated GFP-positive territories obtained by applying a virtual mask to the z stacks acquired by high-resolution confocal microscopy. Quantification of CellROX in the masked GFP-positive 3D projections showed that Tg(fli1a:EGFP)^y5^*nrp1a*^−/-^ embryos had significantly higher CellROX signal within the vasculature compared with control embryos (Figures 2H and 2I). Furthermore, ECs in the intersomitic vessels (ISV) of *nrp1a*^−/-^ knockout embryos were highly positive for CellROX (white arrows, Figure 2H′), suggesting that the migratory ECs forming the ISVs are likely more susceptible to oxidative stress upon NRP1 deletion. These data demonstrate that NRP1 reduces oxidative stress *in vivo* and prompted us to investigate the molecular mechanism by which NRP1 regulates the redox status in ECs.

Because NRP1 downregulation reduces mitochondrial activity (Figures 2A–2D) and induces oxidative stress (Figures 2F–2I), we hypothesized that the increased oxidative stress observed in NRP1-deficient ECs damages the mitochondria and results in reduced mitochondrial activity. Thus, we measured ΔΨ in HDMECs expressing or lacking NRP1 and treated with the mitochondria-targeted antioxidant mitoTEMPO (Figure 2J), which is known to preserve mitochondrial membrane potential in oxidative stress conditions (Dikalova et al., 2010). Strikingly, mitoTEMPO treatment of HDMECs si-NRP1 significantly increased ΔΨ to a level similar to control (Figures 2J and 2K), indicating a recovered mitochondrial membrane potential in NRP1-deficient HDMECs following the mitoTEMPO treatment. These data suggest that ROS induce mitochondrial damage, which results in lowered mitochondrial activity in NRP1-deficient HDMECs.

To further investigate the role of NRP1 in the mitochondria of ECs, we used a mass-spectrometry-based post-translation modification analysis (PTMscan) approach to analyze changes in serine phosphorylation in the proteome of HDMECs transfected with si-NRP1 or si-control. We found that NRP1 downregulation affected serine phosphorylation of several mitochondrial enzymes (Figure S2I, Table S1). Phosphorylation of ATPase family AAA-domain-containing protein 1 and of the precursor of NADH dehydrogenase flavoprotein 3 isoform b was affected. Furthermore, NRP1 knockdown reduced pyruvate dehydrogenase E1 component subunit alpha isoform 2 phosphorylation, which is known to inhibit pyruvate dehydrogenase enzymatic activity (Akhmedov et al., 2012). Strikingly, the mitochondrial-specific ATP-binding cassette (ABC) subfamily B member 8 (ABCB8) was 48 times less phosphorylated in HDMECs lacking NRP1 compared with si-control (Figure S2I, Table S1). The ATP-binding cassette transporter superfamily consists of transmembrane proteins that translocate substrates such as peptides, inorganic anions, amino acids, polysaccharides, proteins, vitamins, and metallic ions across extra- and intracellular membranes (Begicevic and Falasca, 2017). Specifically, ABCB8 is a constituent of the inner mitochondrial membrane recently shown to be involved in mitochondrial iron export (Dean and Allikmets, 2001, Ichikawa et al., 2012). These data indicate that NRP1 modulates mitochondrial function by regulating phosphorylation of key mitochondrial proteins and strongly suggest that NRP1 could regulate mitochondrial iron transport via ABCB8.

### NRP1 Interacts with the ATP-Binding Cassette Protein ABCB8

**Figure 3:**
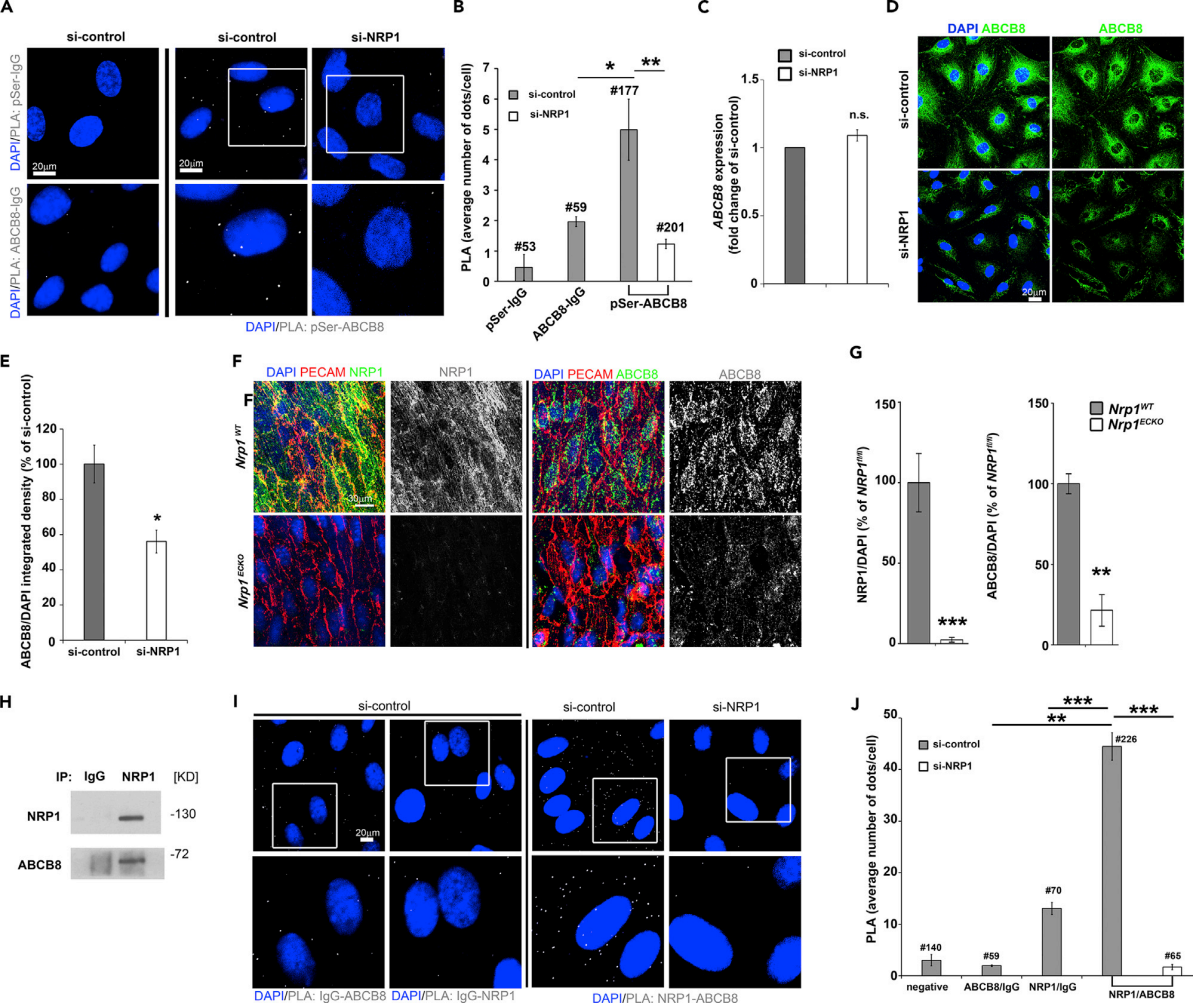
NRP1 Localizes into the Mitochondria and Interacts with ABCB8 (A) PLA for ABCB8 and phosphoserine in HDMECs si-control or si-NRP1; PLA with IgG isotypes was performed as control; scale bar, 20 μm. (B) PLA signal (gray) per cells (mean ± SEM) was measured in a minimum of 177 cells from two independent experiments; # = number of cells analyzed. (C) Quantification of *ABCB8* mRNA by RT-qPCR in HDMECs si-NRP1 or si-control; n = 3. (D) HDEMC si-NRP1 and si-control were stained for ABCB8 (green) and counterstained with DAPI (blue); scale bars, 20 μm. (E) Integrated density of ABCB8 signal was quantified and expressed as percentage relative to si-control (mean ± SEM; n = 3). (F) Aortas of *Nrp1*^fl/fl^ (*Nrp1*^WT^) or *Nrp1*^fl/fl^;Cdh5(PAC)-iCre^ERT2^ (*Nrp1*^ECKO^) littermates injected daily with tamoxifen for 5 days and sacrificed after 1 month from injections were immunostained for NRP1, ABCB8, and PECAM; scale bar, 30 μm. (G) NRP1 and ABCB8 pixel number and intensity were measured in optical z stacks, normalized to DAPI, and expressed as percentage of *Nrp1*^WT^ (mean ± SEM; n ≥ 3 mice each genotype; **p < 0.005, ***p < 0.001, two-way ANOVA). (H) Co-immunoprecipitation of endogenous NRP1 and ABCB8 protein from lysates of HDMECs performed with an anti-NRP1 antibody, followed by immunoblotting for NRP1 and ABCB8 (n = 3). (I) PLA for NRP1 and ABCB8 in HDMECs si-control or si-NRP1. PLA with IgG isotypes was performed as control; scale bar, 20μm. (J) Average PLA signal (gray) per cells (mean ± SEM) was measured in a minimum of 59 cells from four independent experiments. # = number of cells analyzed; *p < 0.05; **p < 0.005, ***p < 0.001; n.s., not significant; Student's t test. See also Figures S1 and S2.

Given that NRP1 loss reduces phosphorylation of ABCB8, we hypothesized that NRP1 regulates iron levels and iron-induced oxidative stress via ABCB8 in ECs. We first confirmed that ABCB8 is expressed in HDMECs and localizes in the mitochondria by co-staining HDMECs for ABCB8 and TOM20 (Figure S2J). We then investigated ABCB8 serine phosphorylation in HDMECs si-NRP1 or si-control by PLA using an anti-phosphoserine and an anti-ABCB8 antibody or control IgG (Figure 3A). In accordance with PTMscan data, the PLA signal detected in HDMECs si-NRP1 was significantly reduced compared with HDMECs si-control (Figure 3B). Because the changes in ABCB8 phosphorylation levels detected by PTMscan and PLA in HDMECs lacking NRP1 could result from a reduction in ABCB8 expression, we investigated whether NRP1 downregulation reduces ABCB8 transcript and protein levels. NRP1 downregulation had no effect on ABCB8 mRNA level (Figure 3C), whereas ABCB8 protein levels were significantly reduced in HDMECs lacking NRP1 compared with control (Figures 3D and 3E), indicating that the reduced levels of ABCB8 phosphorylation could be caused by a decrease in ABCB8 expression. To examine if NRP1 promotes ABCB8 protein expression *in vivo*, we analyzed ABCB8 protein levels in aortas of adult conditional *Nrp1*-null mice lacking (*Nrp1*^WT^) or expressing a tamoxifen-inducible, endothelial-specific Cre transgene (*Nrp1*^ECKO^). NRP1 and ABCB8 staining was performed on whole-mount aortas where ECs were counterstained with PECAM (Figures 3F and S1H). Tamoxifen treatment significantly reduced NRP1 expression in ECs of *Nrp1*^ECKO^ compared with *Nrp1*^WT^ control littermates, indicating that NRP1 was efficiently depleted. Strikingly, ABCB8 expression was significantly reduced in the endothelium of *Nrp1*^ECKO^ compared with *Nrp1*^WT^ littermates (Figures 3F and 3G). Taken together, these data demonstrate that NRP1 regulates ABCB8 protein levels *in vitro* and *in vivo*.

Because ABCB8 is a mitochondria-specific protein and a subcellular pool of NRP1 localizes in the mitochondria, we investigated whether NRP1 forms a protein complex with ABCB8 in HDMECs. Immunoprecipitation analysis performed by pulling down endogenous NRP1 and by probing the immunoprecipitated fraction for NRP1 and ABCB8 showed that NRP1 and ABCB8 form a protein complex (Figure 3H). PLA confirmed the protein-protein interaction observed by co-immunoprecipitation analysis (Figures 3I and 3J). These data further demonstrate that NRP1 localizes in the mitochondria and show that NRP1 interacts with ABCB8; the results also strongly suggest that the formation of this complex promotes ABCB8 function. Furthermore, the data suggest that NRP1 suppresses mitochondrial iron accumulation via ABCB8, thus preventing iron-dependent oxidative stress.

### NRP1 Regulates Iron Homeostasis and Suppresses Iron-Dependent Oxidative Stress in ECs

**Figure 4:**
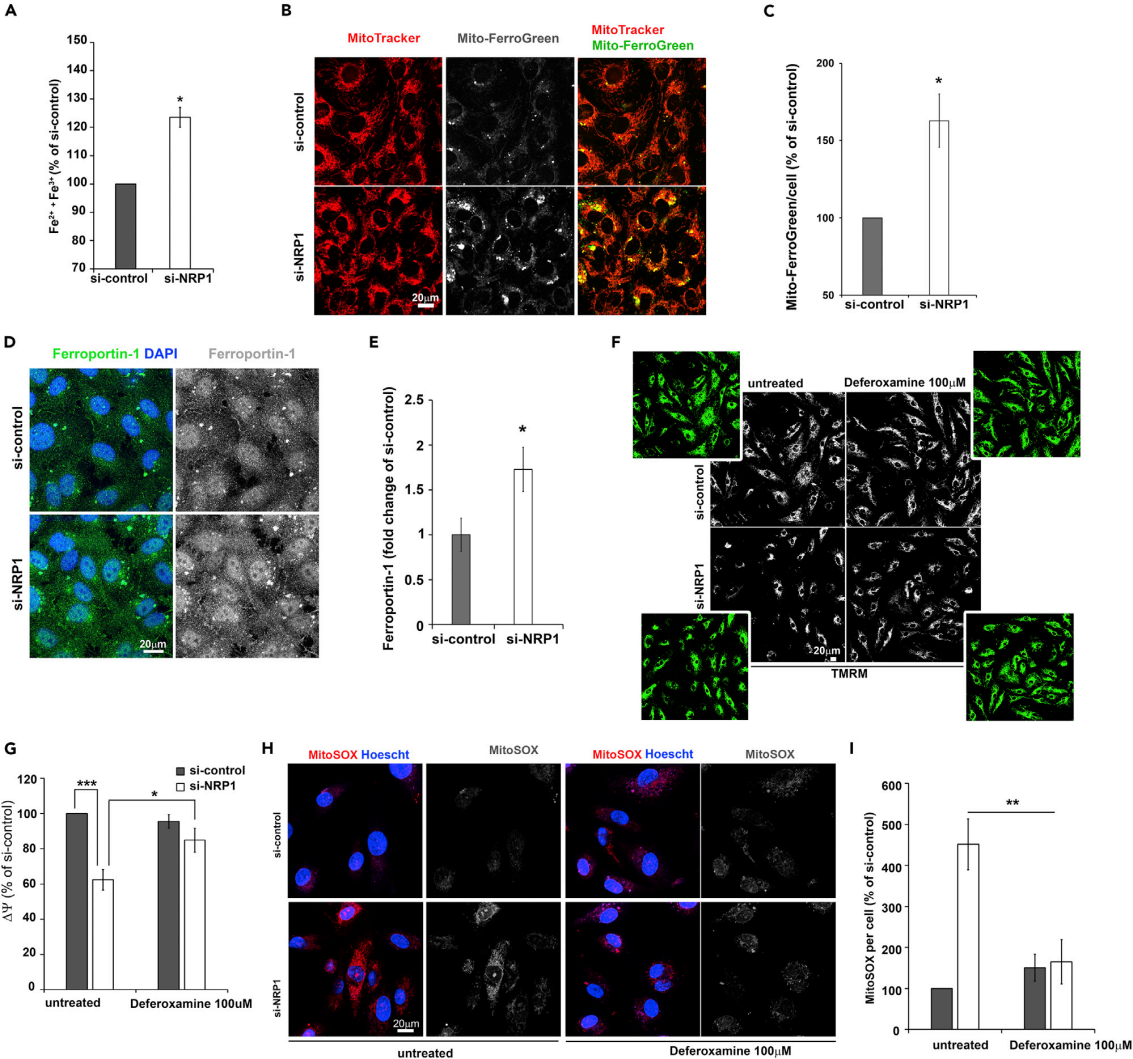
NRP1 Regulates Iron Homeostasis And Iron-Dependent Oxidative Stress (A) Intracellular total iron (Fe^2+^ plus Fe^3+^) of HDMECs si-control or si-NRP1 expressed as percentage relative to si-control (mean ± SEM; n = 3). (B) HDMECs si-NRP1 or si-control were incubated with 5 μM Mito-FerroGreen (gray) and 300 nM MitoTracker Deep Red FM (red); scale bar, 20 μm. (C) Mito-FerroGreen fluorescent signal per cell was quantified and expressed as percentage relative to si-control (mean ± SEM; n = 3). (D) HDEMCs si-NRP1 or si-control were stained for ferroportin-1 (green or gray) and counterstained with DAPI (blue); scale bar: 20 μm. (E) Integrated density of ferroportin-1 was normalized to DAPI and expressed as fold change of si-control (mean ± SEM; n = 4). (F) HDMECs treated with deferoxamine 100 μM for 24 hr after 48 hr from transfection with si-NRP1 or si-control were incubated with 100 nM TMRM (gray); scale bars, 20 μm. (G) Integrated density of the TMRM signal normalized to cell number determined by manual counting using high-contrast images (panel F, green images) and expressed as percentage relative to si-control (mean ± SEM; n = 4). (H) HDMECs si-control or siNRP1 untreated or treated with deferoxamine 100 μM for 24 hr were incubated with 5 μM MitoSOX (red), counterstained with Hoescht 33342 (blue); scale bar: 20 μm. (I) Integrated density of the MitoSOX signal expressed as percentage relative to untreated si-control (mean ± SEM; n = 4). *p < 0.05, ***p < 0.001; Student's t test. See also Figure S3.

To investigate whether NRP1 regulates iron homeostasis, we measured intracellular ferrous (Fe^2+^) and ferric (Fe^3+^) content in HDMECs lacking or expressing NRP1 using a ferene S-based colorimetric assay (Bai et al., 2017, Ichikawa et al., 2012). Intracellular iron was significantly higher in HDMECs downregulated for NRP1 expression compared with control (Figure 4A), indicating that NRP1 loss results in iron accumulation. To further investigate whether NRP1 downregulation induces mitochondrial iron accumulation we measured Fe^2+^ in HDMECs expressing or lacking NRP1 expression by incubating ECs with Mito-FerroGreen, a mitochondria-specific iron probe that becomes fluorescent when reacting with labile mitochondrial Fe^2+^ (Hirayama et al., 2018). Co-staining of HDMECs with Mito-FerroGreen and MitoTracker confirmed a complete mitochondrial localization of Mito-FerroGreen in HDMECs (Figure 4B). In agreement with the increased intracellular iron observed in HDMECs si-NRP1 (Figure 4A), NRP1 downregulation induced a significant increase in mito-FerroGreen staining, indicating that loss of NRP1 expression induces mitochondrial iron accumulation in ECs (Figures 4B and 4C).

Then we investigated the expression of iron transporters known to modulate intracellular iron levels. No change was observed in the mRNA levels and protein expression of transferrin receptor 1, which mediates cellular iron uptake (Figures S3A and S3B). Expression of ferroportin-1, which regulates EC iron efflux, was significantly increased at the protein level (Figure 4D), but unchanged at the mRNA level (Figure S3A), following NPR1 downregulation. Interestingly, NRP1 downregulation significantly increased the expression of mitochondria-specific iron importer Mitoferrin-1 but had no effect on Mitoferrin-2 expression (Figure S3C). Taken together these data suggest that loss of NRP1 induces iron accumulation by dysregulating iron influx-efflux balance in ECs.

To investigate whether iron accumulation reduces mitochondria activity in HDMECs downregulated for NRP1 expression, we measured the mitochondrial membrane potential ΔΨ in HDMECs expressing or lacking NRP1 and treated with the iron chelator deferoxamine (Figure 4F). As previously shown (Figures 2A–2D), NRP1 downregulation significantly decreased the ΔΨ-dependent TMRM staining (Figures 4F and 4G). Strikingly, deferoxamine treatment of HDMECs si-NRP1 rescued the TMRM staining to a level comparable to HDMECs si-control (Figures 4F and 4G), indicating that iron-mediated ROS production causes mitochondrial damage, which results in reduced ΔΨ. Similarly, deferoxamine treatment rescued ΔΨ in HUVECs lacking NRP1 (Figures S3D and S3E). Thus we investigated whether iron induces ROS production by measuring mitochondrial ROS in HDMECs si-NRP1 treated with the iron chelator deferoxamine. As previously shown (Figures 2E and 2F), MitoSOX live-stained HDMECs lacking NRP1 expression have significantly higher mitochondrial superoxide level compared with control cells (Figures 4H and 4I). Importantly, deferoxamine treatment normalized superoxide levels in HDMECs si-NRP1 to levels similar to control cells (Figures 4H and 4I). Taken together these data demonstrate that lack of NRP1 induces iron-dependent oxidative stress, which causes a decrease of the mitochondrial activity and increases mitochondrial ROS levels.

### NRP1 Regulates Iron Homeostasis via ABCB8

**Figure 5:**
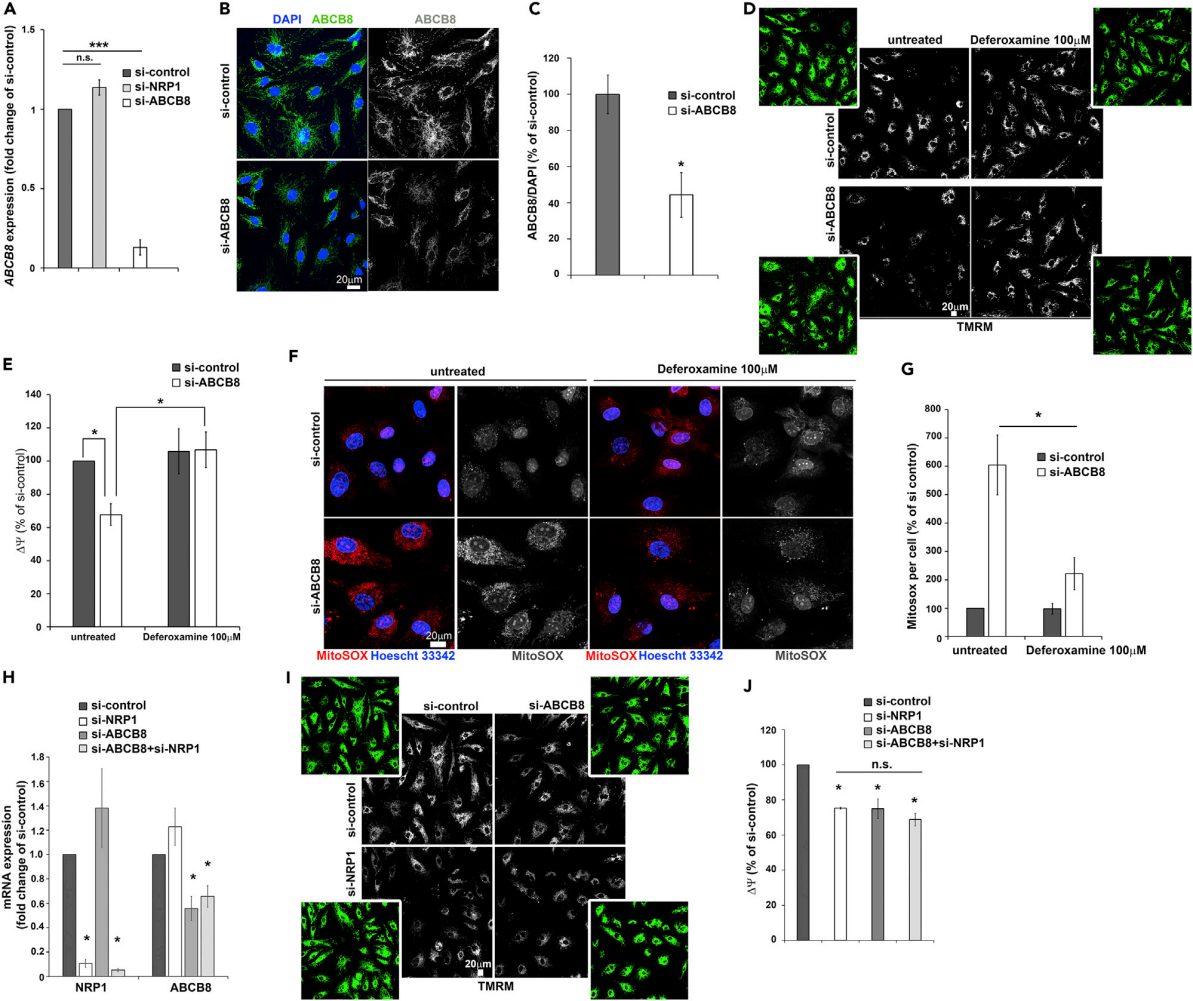
NRP1 Regulates Iron-Dependent Oxidative Stress via ABCB8 (A) Quantification of *ABCB8* mRNA by RT-qPCR in HDMECs si-ABCB8, si-NRP1, or si-control. Transcripts levels were expressed as fold change of si-control (mean ± SEM; n = 3). (B) Immunostaining for ABCB8 (green) in HDMECs si-ABCB8 or si-control counterstained with DAPI (blue); scale bar, 20 μm. (C) ABCB8 integrated density in optical z stacks was normalized to DAPI integrated density and expressed as percentage relative to si-control (mean ± SEM; n = 3). (D) HDMECs treated with deferoxamine 100 μM for 24 hr, 48 hr after transfection with si-ABCB8 or si-control were incubated with 100 nM TMRM (gray); scale bar, 20 μm. (E) Integrated density of the TMRM signal was normalized to cell number determined by manual counting using high-contrast images (Figure 5D, green panels) and expressed as percentage (mean ± SEM; n = 4). (F) HDMECs si-ABCB8 or si-control were incubated with 5 μM MitoSOX (red) and counterstained with Hoescht 33342 (blue); n = 4; scale bar, 20 μm. (G) Integrated density of the MitoSOX signal was quantified as percentage of si-control (mean ± SEM; n = 4). (H) Quantification of NRP1 and ABCB8 mRNA by RT-qPCR in HDMECs single or double transfected with si-NRP1 and si-ABCB8 or si-control (mean ± SEM; n = 3). (I) HDMECs single or double transfected with si-ABCB8 and si-NRP1 or si-control were incubated with 100 nM TMRM (gray); scale bar, 20 μm. (J) Integrated density of the TMRM signal was normalized to cell number determined by manual counting using high-contrast images (panel I, green images) and expressed as percentage (mean ± SEM; n = 3). *p < 0.05, ***p < 0.001; n.s., not significant; Student's t test. See also Figure S3.

To further investigate whether NRP1 regulates iron-dependent oxidative stress via ABCB8, we downregulated ABCB8 expression using siRNA in HDMECs (si-ABCB8). *ABCB8* mRNA, immunostaining, and immunoblotting analyses showed that HDMECs transfected with si-ABCB8 had significantly reduced ABCB8 expression (Figures 5A–5C and S3F). Then we investigated whether ABCB8 downregulation decreases mitochondrial ΔΨ similarly to NRP1 knockdown and whether deferoxamine treatment could normalize ΔΨ in HDMECs and HUVECs knockdown for ABCB8. HDMECs and HUVECs lacking ABCB8 expression showed reduced TMRM staining compared with control (Figures 5D, 5E, S3G, and S3H) similarly to NRP1 downregulation (Figures 2B–2D, 4F, and 4G). Strikingly, deferoxamine treatment restored ΔΨ of HDMECs and HUVECs si-ABCB8 to levels comparable with those of control cells (Figures 5D, 5E, S3G, and S3H). These data indicate that lack of ABCB8 or NRP1 similarly induces an iron-mediated decrease in mitochondrial ΔΨ and strongly suggest that NRP1 and ABCB8 act on the same signaling pathway. Because ABCB8 downregulation induces iron-mediated ROS production in cardiomyocytes and melanoma cells (Ichikawa et al., 2012, Ichikawa et al., 2014), we investigated whether ABCB8 regulates iron-dependent oxidative stress by measuring mitochondrial superoxide level in HDMECs expressing or lacking ABCB8 and treated with deferoxamine. MitoSOX live-staining analysis showed that ABCB8-deficient HDMECs had significantly higher MitoSOX staining compared with si-control (Figures 5F and 5G), similarly to NRP1 downregulation (Figures 2E and 2F), suggesting that both NRP1 and ABCB8 regulate iron-mediated ROS production in ECs. Accordingly, deferoxamine completely inhibited superoxide production in HDMECs si-ABCB8 (Figures 5F and 5G). These data demonstrate that ABCB8 downregulation induces iron-dependent ROS production in ECs and suggest that NRP1 regulates these processes through ABCB8.

To further investigate this hypothesis, we measured mitochondrial ΔΨ and ROS in HDMECs with a simultaneous knockdown for ABCB8 and NRP1. Downregulation of both NRP1 and ABCB8 (Figure 5H) had no additive effect in inhibiting mitochondrial ΔΨ compared with HDEMCs lacking NRP1 or ABCB8 only (Figures 5I and 5J), suggesting that NRP1 and ABCB8 act on the same cellular pathway.

### NRP1 and ABCB8 Loss Induces Iron-Dependent Premature Senescence in ECs

**Figure 6:**
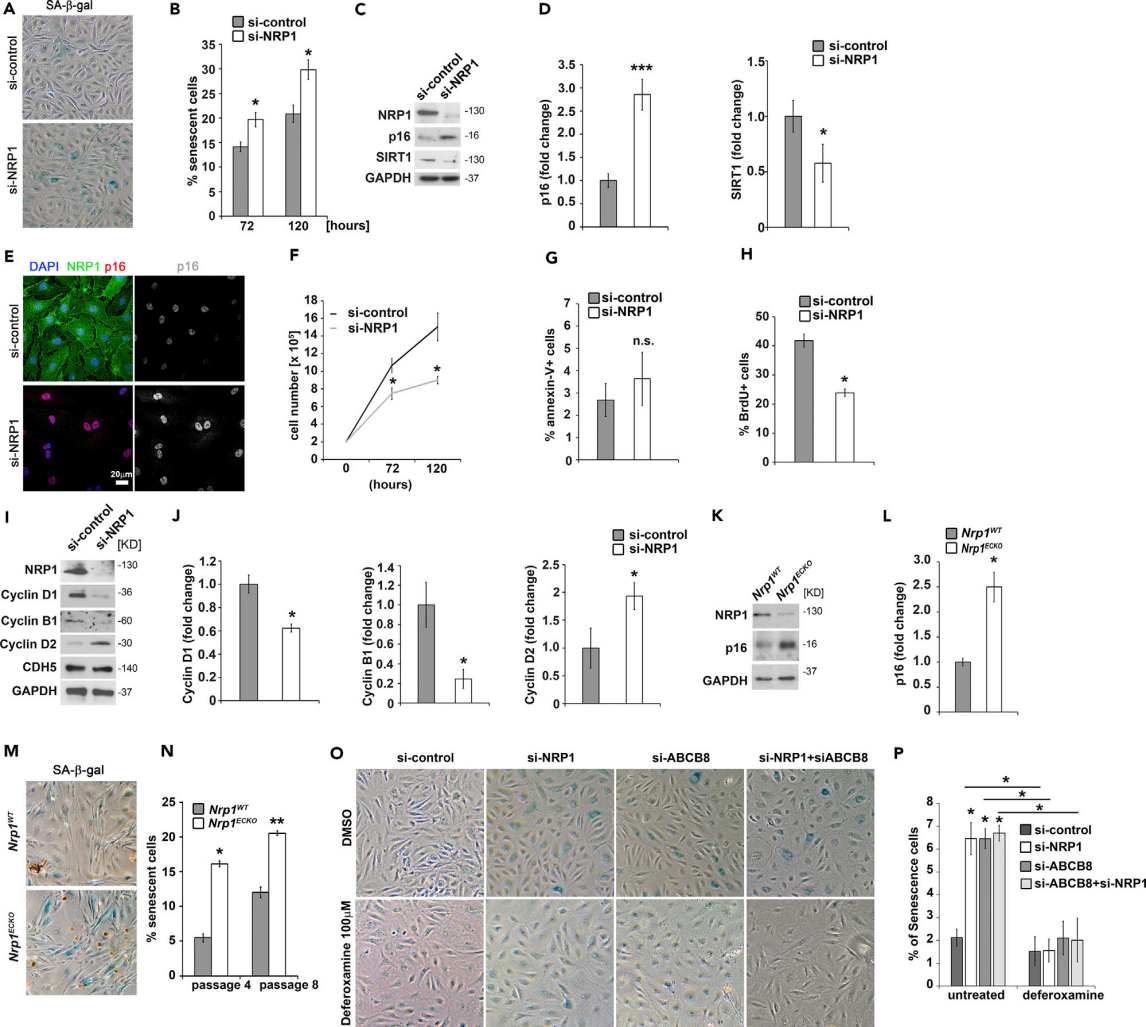
NRP1 Suppresses Iron-Dependent Premature Senescence via ABCB8 (A) SA-β-gal staining in HDMECs si-control or si-NRP1 72 and 120 hr after transfection. (B) Quantification of SA-β-gal-positive cells (mean ± SEM; n = 3). (C) Representative immunoblots for NRP1, p16, and SIRT1 of HDMECs si-control or si-NRP1. (D) Quantification of p16 expression level as pixel intensity relative to GAPDH pixel intensity (mean ± SEM; n = 4). (E) Immunofluorescence for NRP1 (green) and p16 (red) together with DAPI staining; scale bar, 20 μm; n = 2. (F) Cell counting assay at 72 and 120 hr after transfection in HDMECs si-control or si-NRP1 (mean ± SEM; n = 3). (G) FACS analysis of annexin-V-positive cells in HDMECs si-control or si-NRP1 (mean ± SEM; n = 3). (H) Analysis of bromodeoxyuridine (BrdU)-positive cells in HDMECs si-NRP1 or si-control (mean ± SEM; n = 3). (I) Representative immunoblotting for cyclin D1, cyclin B1, cyclin D2, and CHD5 with GAPDH serving as a loading control of HDMECs si-control or si-NRP1. (J) Quantification of the indicated protein expression levels as pixel intensity relative to GAPDH pixel intensity (mean ± SEM; n = 4). (K) Representative immunoblotting for the indicated proteins of MLECs from *Nrp1*^ECKO^ and *Nrp1*^WT^ littermates. (L) Quantification of p16 total levels as pixel intensity relative to GAPDH (mean ± SEM; n = 4). (M) SA-β-gal assay of MLECs from *Nrp1*^ECKO^ and *Nrp1*^WT^ littermates. (N) Quantification of SA-β-gal-stained MLECs after four and eight tissue culture passages. (O) SA-β-gal staining in HDMECs double transfected for 24 hr with si-NRP1, si-ABCB8, or si-control and treated with deferoxamine 100 μM for 48 hr. (P) Quantification of SA-β-gal-positive cells (mean ± SEM; n = 3). *p < 0.05, ***p < 0.001; n.s., not significant; Student's t test.

Cellular senescence can be prematurely induced by telomere-independent mechanisms, which include excessive oxidative stress and mitochondrial dysfunction (Erusalimsky, 2009). Thus we hypothesized that loss of NRP1 could result in premature senescence in ECs, and we investigated whether NRP1-deficient ECs presented the hallmarks of senescence. Cellular senescence is characterized by elevated senescence-associated β-galactosidase (SA-β-gal) activity (Dimri et al., 1995, Ota et al., 2008, Warboys et al., 2014), increased p16 levels (Chen et al., 2006, Song et al., 2008), reduction of SIRT1 expression (Ota et al., 2008), and reduced proliferation. NRP1-deficient HDMECs showed an increased number of SA-β-gal-positive cells compared with control (Figures 6A and 6B) alongside increased p16 levels and reduced SIRT1 expression (Figures 6C–6E). Furthermore, cell counting analysis showed a reduced cell growth rate of NPR1-downregulated HDMECs compared with control under normal growth conditions (Figure 6F). Because it has been previously reported that NRP1 is required to mediate anti-apoptotic signals in serum-starved ECs (Wang et al., 2007), we measured the apoptosis marker annexin-V (Koopman et al., 1994) by fluorescence-activated cell sorting (FACS) to determine whether the reduced cell growth observed in NRP1-deficient ECs was due to increased apoptosis also in normal growth conditions. The number of cells undergoing apoptosis was similar in HDMECs si-NRP1 and si-control (Figure 6G), suggesting that the reduced cell growth observed in normal growth conditions is not caused by increased apoptosis. Importantly, FACS analysis of bromodeoxyuridine-labeled HDMECs si-NRP1 or si-control showed that NRP1 deficiency significantly reduced the number of proliferating cells in HDMECs si-NRP1 compared with controls (Figure 6H). Thus NRP1 loss reduces EC proliferation without inducing apoptosis in normal growth conditions.

Because EC proliferation was affected by NRP1 loss, we investigated whether the expression of cyclins was affected by NRP1 knockdown. In agreement with the reduced cell growth, immunoblotting analysis showed reduced cyclin D1 and cyclin B1 levels and a concomitant increase in cyclin D2 expression in HDMECs si-NRP1 compared with si-control (Figures 6I and 6J). Thus loss of NRP1 affects cyclin expression and therefore cell cycle progression in ECs.

To further investigate the role of NRP1 in EC senescence, we measured p16 protein level and the number of SA-β-gal-positive cells also in primary mouse lung endothelial cells (MLECs) isolated from mice genetically deficient for NRP1 in ECs (*Nrp1*^ECKO^) and from control littermates (*Nrp1*^WT^). *Nrp1*^ECKO^ MLECs showed significantly higher p16 protein expression (Figures 6K and 6L) and increased number of SA-β-gal-positive cells (Figures 6M and 6N) compared with MLECs isolated from *Nrp1*^WT^ littermates. Taken together these data demonstrate that loss of NRP1 induces premature senescence.

We then investigated whether by regulating iron homeostasis the NRP1-ABCB8 pathway protects ECs from premature senescence. SA-β-gal staining showed that NRP1 or ABCB8 downregulation similarly increased the number of SA-β-gal-positive HDMECs (Figures 6O and 6P). Furthermore, treatment of HDMECs lacking both NRP1 and ABCB8 with deferoxamine reduced SA-β-gal-positive cells to a level similar to untreated si-control (Figures 6O and 6P). These data show that NRP1-ABCB8 pathway suppresses iron-induced ECs senescence.

## Discussion

Despite oxygen availability, ECs mainly rely on anaerobic glycolysis to meet their energy demand and to produce ATP (De Bock et al., 2013). Thus although mitochondria provide a limited contribution to energy production in ECs, they are essential for EC function, because altering mitochondrial dynamics contributes to endothelial dysfunction (Shenouda et al., 2011). Accordingly, ECs lacking MFN1 show reduced VEGF-induced angiogenesis and inhibited AKT-dependent activation of endothelial nitric oxide synthase (Lugus et al., 2011). Here we show that NRP1 regulates mitochondrial activity and dynamics. ECs lacking NRP1 fail to form the complex mitochondrial network observed in control ECs and show a reduced level of MFN1. Mitochondrial dysfunction plays a role in the onset of cellular senescence, and preventing mitochondrial dysfunction delays replicative senescence in human primary cells (Passos et al., 2007). Furthermore, excessive production of ROS is associated with mitochondrial dysfunction and with the induction of p16INK4A-dependent senescence (Davalli et al., 2016).

Cellular senescence limits the capacity of tissues to undergo repair by cell proliferation and thereby accelerates age-related degenerative diseases such as sarcopenia (Kudryashova et al., 2012), osteoarthritis (Price et al., 2002), pulmonary fibrosis (Tsuji et al., 2010), and Alzheimer disease (Bhat et al., 2012). In the aging vasculature, senescent ECs accumulate in atherosclerotic lesions and likely contribute to disease progression by creating a pro-inflammatory and pro-thrombotic environment (Minamino et al., 2002, Vasile et al., 2001, Warboys et al., 2014). Here we show that ECs lacking NRP1 show the hallmarks of cellular senescence, including high levels of p16 and decreased SIRT1 expression alongside deregulated cyclin expression, reduced proliferation, as well as an increased SA-β-gal staining.

**Figure 7:**
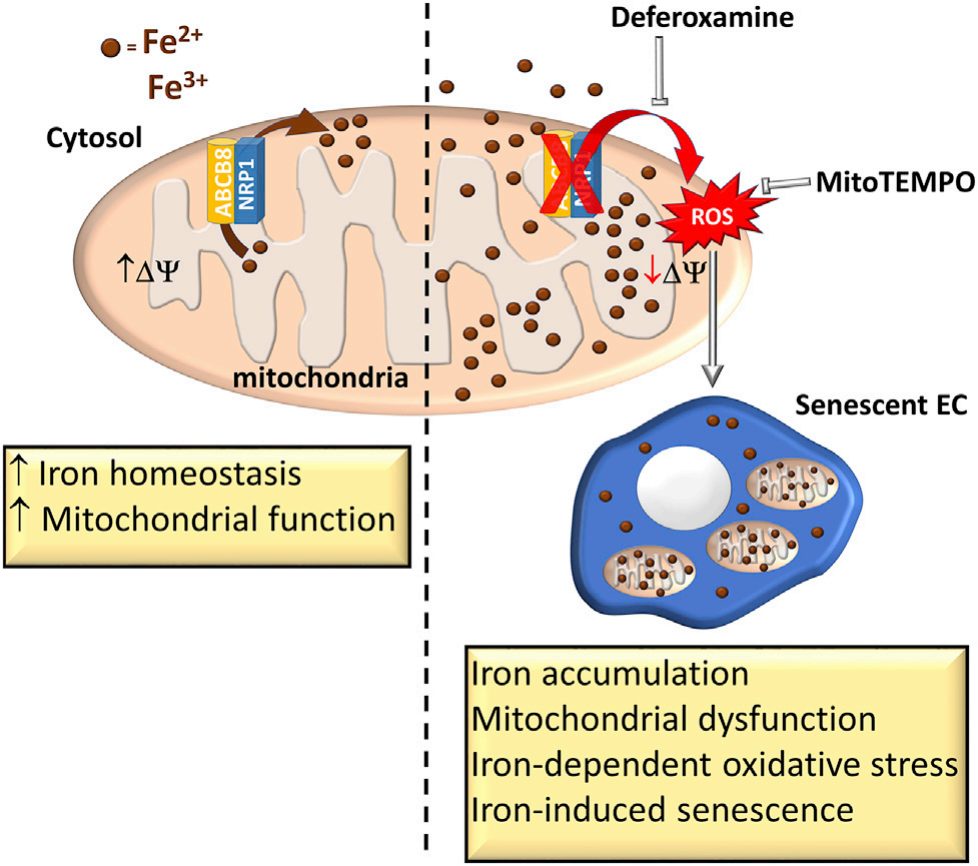
Schematic Representation of the NRP1-ABCB8 Pathway in Regulating Iron Homeostasis, Iron-Dependent Oxidative Stress, and Cellular Senescence

Furthermore, we show that NRP1 prevents mitochondrial superoxide production and EC senescence by promoting iron homeostasis via the mitochondrial transporter ABCB8 (Figure 7). As a pool of NRP1 localizes in the mitochondria and interacts with the mitochondrial inner membrane transporter ABCB8, the NRP1-ABCB8 complex likely forms in the inner mitochondrial membrane compartment. NRP1 downregulation reduces ABCB8 protein expression and phosphorylation. The ABCB8 reduction observed in NRP1-deficient ECs could be explained on one hand by the reduced mitochondria content, and on the other hand by the disruption of the ABCB8/NRP1 complex, which could promote localization of ABCB8 in the mitochondria or prevent ABCB8 protein degradation. Importantly, because HDMECs si-NRP1 show a partial reduction in ABCB8 protein content but PTMscan and PLA analyses display an almost complete inhibition in ABCB8 phosphorylation, our data suggest that the decreased phosphorylation of ABCB8 observed in HDMECs si-NRP1 could be caused by a combination of the reduced ABCB8 protein level with an additional mechanism that could inhibit ABCB8 phosphorylation in NRP1-deficient ECs.

Treatment with the iron chelator compound deferoxamine restores the mitochondrial membrane potential of NRP1-deficient HDMECs and HUVECs and abrogates mitochondrial superoxide production. Deferoxamine has been shown to promote blood flow recovery in a hindlimb ischemia mouse model (Ikeda et al., 2011) and to increase hypoxia-inducible transcription factor 1, VEGFA, and COX-2 levels in ischemic skeletal muscle and in macrophages (Asikainen et al., 2005, Woo et al., 2006). A role for iron has also been proposed in the pathogenesis of atherosclerosis (Kiechl et al., 1997, Lee et al., 1999), and deferoxamine treatment improved nitric oxide-mediated, endothelium-dependent vasodilation in patients with coronary artery disease (Duffy et al., 2001).

By showing that deferoxamine restores mitochondrial membrane potential and reduces ECs senescence in NRP1-deficient ECs (Figure 7), our data raise the possibility that targeting iron metabolism could be a promising strategy to prevent ECs senescence and preserve endothelial function.

The function of NRP1 in regulating mitochondrial homeostasis and iron metabolism independently of VEGFR2 signaling expands the current model, which suggests that NRP1 promotes several signals in ECs and is not acting exclusively as a VEGF co-receptor (Aspalter et al., 2015, Hu et al., 2007, Raimondi et al., 2014, Raimondi et al., 2015, Raimondi et al., 2016). Because the NRP1-ABCB8 pathway is essential to prevent iron-induced mitochondrial dysfunction, our data suggest that in addition to its well-characterized role in angiogenesis, arteriogenesis (Fantin et al., 2014, Kawasaki et al., 1999, Lanahan et al., 2013, Raimondi, 2014), and vascular permeability (Fantin et al., 2017, Roth et al., 2016), NRP1 regulates a fundamental pathway that promotes ECs homeostasis.

In ECs, mitochondria function as a metabolic hub (Davidson, 2010, De Bock et al., 2013, Quintero et al., 2006). Mitochondria are involved in the production of aspartate and glutamate needed for deoxynucleotide synthesis (Rohlenova et al., 2017, Schoors et al., 2015), in the regulation of glutamine metabolism whose inhibition causes vessel-sprouting defects due to impaired proliferation and migration (Huang et al., 2017), and in iron metabolism. Because Fe/S clusters and heme's precursor biosynthesis occurs in the mitochondria, physiological mitochondrial iron levels are maintained through the activity of mitochondria-specific iron importers and exporters. Mitoferrin-1 and mitoferrin-2 mediate iron uptake in fibroblasts, murine embryonic stem cells, and erythroleukemia cells (Chen et al., 2010, Paradkar et al., 2009), whereas recent evidence shows that the mitochondrial inner membrane transporter ABCB8 regulates ionic iron export and protects mitochondria from iron overload in melanoma cells and cardiomyocytes (Ardehali et al., 2005, Ichikawa et al., 2012, Ichikawa et al., 2014). Accordingly, mouse mutants lacking ABCB8 expression in cardiomyocytes develop cardiomyopathy due to increased mitochondrial iron, which results in iron-dependent ROS production and mitochondrial dysfunction (Ichikawa et al., 2012). Our data show that ABCB8 plays a key role in iron homeostasis also in ECs. Furthermore, our data suggest a profound role of NRP1 in regulating mitochondrial iron because its downregulation simultaneously increases the mitochondrial importer mitoferrin-1 transcript and decreases ABCB8 protein level. As the data presented here and in previous reports (Ichikawa et al., 2012, Ichikawa et al., 2014) show that ABCB8 prevents iron-mediated mitochondrial damage, it is possible that by decreasing ABCB8-dependent iron export, NRP1 loss impairs mitochondrial function and therefore iron metabolism, thus triggering a compensatory response to increase transcription of the mitochondrial iron importer mitoferrin-1 to restore iron metabolism and of ferroportin-1 to maintain physiological intracellular iron levels.

Our data suggest that promoting NRP1-dependent signaling could be particularly relevant in age-related diseases characterized by increased oxidative stress or by deregulation of iron metabolism such as atherosclerosis, vascular dementia, and Alzheimer disease. Oxidative stress and iron-mediated free radical production mediate neuronal death and gliosis. Furthermore, oxidative stress induces endothelial dysfunction in a model of cerebral ischemia (Jin et al., 2008). Importantly, patients with Alzheimer or Parkinson diseases have higher iron content, which correlates with increased ROS levels in areas of the brain prone to neurodegeneration (Manoharan et al., 2016). Recent evidence suggests that the neurodegenerative process observed in neurodegenerative diseases is initiated by blood-brain barrier dysfunction, caused by aging and cardiovascular conditions, resulting in increased oxidative stress, dysregulation of nitric oxide, inflammation, and altered paracellular permeability (Oakley and Tharakan, 2014, Sweeney et al., 2018). Whether NRP1 expression or activity is modulated in these diseases is unknown, and our work suggests that pathological downregulation of NRP1 could have detrimental consequences on EC homeostasis.

In conclusion, our data demonstrate an unexpected function of NRP1, which acts as a key regulator of mitochondria and iron homeostasis in ECs by suppressing iron accumulation and iron-dependent oxidative stress via ABCB8, independently of VEGF signaling. Our work suggests that the NRP1-ABCB8 pathway could protect ECs function from iron-dependent ROS-induced endothelial dysfunction and have a protective role in age-related diseases.

### Limitation of the Study

To identify the NRP1-ABCB8 pathway and to investigate its role in ECs, we used an *in vitro* approach that relied on siRNA-mediated transient downregulation of NRP1 and ABCB8. This approach allows neither the long-term effects of NRP1 or ABCB8 downregulation in ECs nor the role of the NRP1/ABCB8 pathway in endothelial function *in vivo* to be studied. Although we have established that genetic manipulation of NRP1 expression prevents oxidative stress in a zebrafish model and protects ECs from senescence in an *ex vivo* model, whether the NRP1-ABCB8 pathway plays a role in physiological or pathological angiogenesis, or in homeostatic EC functions such as nitric oxide synthase activation, vascular permeability, and leukocyte-EC adhesion remains to be addressed. Furthermore, the mechanisms that modulate NRP1 expression in pathological contexts, such as neurodegenerative disorders or ischemia, are currently unclear and would need to be further investigated.

## Methods

All methods can be found in the accompanying Transparent Methods supplemental file.

## Supporting Citations

The following references appear in the Supplemental Information: Lawson and Weinstein, 2002, Stokes et al., 2012, Wang et al., 2010, Westerfield, 1995.

## Supplemental Information

Document S1. Transparent Methods and Figures S1–S3

Table S1. PTMscan phosphoSerine Dataset of HDMECs si-NRP1 or si-Control, Related to Figures 3 and S2
