# Supplementary material for: Neuropilin-1 Controls Endothelial Homeostasis by Regulating Mitochondrial Function and Iron-Dependent Oxidative Stress
Source: iScience. 2018 Dec 11;11:205–23. doi: 10.1016/j.isci.2018.12.005 (PMC6327076; doi:10.1016/j.isci.2018.12.005)
Supplement: Document S1. Transparent Methods and Figures S1–S3 [file mmc1.pdf]

ISCI, Volume *11*

Supplemental Information

Neuropilin-1 Controls Endothelial Homeostasis by Regulating Mitochondrial Function and Iron-Dependent Oxidative Stress

Theo Issitt, Emy Bosseboeuf, Natasha De Winter, Neil Dufton, Gaia Gestri, Valentina Senatore, Anissa Chikh, Anna M. Randi, and Claudio Raimondi

Supplemental Information:

Transparent Methods

Cell culture and transfection:

Human microvascular dermal ECs (HDMECs) were cultured in MV2 media with supplements (Promocell, UK). HUVECs were cultured in EGM-2 (Promocell, Germany). Primary MLECs were isolated from mice between 1 and 2 months of age by magnetic-activated cell sorting (MACS) with PECAM (BD, UK) and ICAM2 (BD, UK). MLECs were cultured on 10 μg/ml FN in DMEM-GlutaMAX supplemented with 20% FBS, nonessential amino acids (Life Technologies, UK), and ECGS (Sigma-Aldrich, UK). HDEMCs and HUVECs were cultured for up to five passages and transfected with Lipofectamine RNAiMAX transfecting agent (Thermofisher, UK). The following siRNAs were used to transfect HDMECs and HUVECs: SMARTpool siRNA targeting NRP1 (Dharmacon, USA; cat#: L-019484-00-0020); sequence 1: CGAUAAAUGUGGCGAUACU; antisense: AGUAUCGCCACAUUUAUCG; sequence 2: GGACAGAGACUGCAAGUAU; antisense: AUACUUGCAGUCUCUGUCC; sequence 3: GUAUACGGUUGCAAGAUAA Antisense: UUAUCUUGCAACCGUAUAC; sequence 4: AAGACUGGAUCACCAUAAA antisense: UUUAUGGUGAUCCAGUCUU; SMARTpool siRNA targeting VEGFR2 (Dharmacon, USA; cat# L-003148-00-0010); sequence 1: GGGCAUGUACUGACGAUUA Antisense: UAAUCGUCAGUACAUGCCC; sequence 2: CUACAUUGUUCUUCCGAUA Antisense: UAUCGGAAGAACAAUGUAG; sequence 3: GCGAUGGCCUCUUCUGUAA; Antisense: UUACAGAAGAGGCCAUCGC sequence 4: GGAAAUCUCUUGCAAGCUA; Antisense: UAGCUUGCAAGAGAUUUCC; SMARTpool siRNA targeting ABCB8 (Dharmacon, USA; cat#: L-007306-02-0010; Sequence 1: CAACACGGUCGUCGGUGAA; antisense: UUCACCGACGACCGUGUUG; sequence 2: UCACCUUCUUUGACGCCAA; Antisense: UUGGCGUCAAAGAAGGUGA: sequence 3: AACGGGAAGAGGAGCGCUA Antisense: UAGCGCUCCUCUUCCCGUU; sequence 4: GCAUUGUCGUCAUGGCCGA; antisense: UCGGCCAUGACGACAAUGC; Silencer® negative control siRNA (Life Technology, UK; cat# AM4635). In some experiments, HDMECs were transfected with Hs NRP1 7 FlexiTube siRNA single sequence (Cat. SI02663213) or Hs NRP1 8 FlexiTube siRNA (Cat. SI02663213) from Qiagen, UK. In some experiments, cells were double transfected with both siNRP1 and siABCB8. The amount of siRNA was

maintained identical among the different single and double transfections. In some experiments, ECs were treated with mitoTEMPO 10µM (Sigma Aldrich, UK) or Deferoxamine (100 µM) (Sigma Aldrich, UK) for 24 hours in growth media.

Animals:

All animal procedures were performed in accordance with institutional and UK Home Office guidelines. Lung endothelial cells were isolated from C57/Bl6 wild type mice (Charles River Laboratories, UK), and mice carrying two floxed conditional null Nrp1 alleles (*Nrp1*$^{fl/fl}$) combined with *Pdgfb-iCre*^{ERT2}*-Egfp* with codon-improved Cre on a C57/Bl6 background (Gu et al., 2003; Raimondi et al., 2014). Aortas were isolated from B6.129 (SJL)-*Nrp1*^{tm2Ddg}/J(Gu et al., 2003) (Charles River Laboratories, UK) combined with C57/Bl6 *Cdh5(PAC)-iCre*^{ERT2} with codon-improved Cre (Wang et al., 2010). Zebrafish lines Tg(fli1a:egfp)y5 (Lawson and Weinstein, 2002) and nrp1a sa1485 (generated by the Sanger Centre Zebrafish Mutation Project) were maintained and bred according to standard procedures (Westerfield, 1995). Ethical approval for zebrafish experiments was obtained from the Home Office UK under the Animal Scientific Procedures Act 1986.

For tamoxifen-induction of CRE-mediated recombination, 0.5 mg of tamoxifen (Sigma Aldrich, UK) dissolved in peanut oil to 2 mg/ml was administered via intraperitoneal injection at the indicated times to generate *Nrp1*^{ECKO} and *Nrp1*^{WT} mice.

Gene expression:

mRNA was collected using the RNAeasy system (Qiagen, UK) and cDNA was prepared using Superscript III reverse transcription (Life Technology, UK) and amplified with SYBR Green PCR reagent (Quantabio, USA) and the following oligonucleotide primers:

NRP1, 5′-GAAAAATCGAATGCTGAT-3′ and 5′-AATCCGGGGGACTTTATCAC-3′; VEGFR2, 5′-AGATGGTGTAACCCGGAGTG-3′ and 5′-ACATGTCAGCGTTTGAGTGG-3′; ABCB8, 5'-AGTACTCTGATGGCTACCGC-3' and 5'-CAGAGGTGGGGATGCTTACT-3'; HO-1, 5′-TTCTATCACCCTCTGCCT-3 and 5′-CCTCTTCACCTTCCCCAACA-3'; SOD1, 5'-GGATGAAGAGAGGCATGTTGGAGAC-3' and 5'-GTCTTTGTACTTTCTTCATTTCCACC-3'; SOD2, 5'-AGCAGTGGAATAAGGCCTGT-3' and 5'-CAAAGGGGAGTTGCTGGAAG-3';

TRF1, 5'-CAGTTGGAGTGCTGGAGACT-3' and 5'-AGCGTATACAACAGTGGGCT-3'; Ferroportin-1, 5'-TACTTGTGCCTCCCAGATGG-3' and 5'-ATGGAACCACTCAGTCCCTG-3'; Mitoferrin-1, 5'-CCAGATCCCAAAGCCCAGTA-3' and 5'-CCATACTCCCAGCTATCCCG-3'; Mitoferrin-2, 5'-CCACGCCCTTTATTTTGCCT-3' and 5'-TCCTCTGCTTGACCACTTCC-3'; GAPDH, 5'-CAAGGTCATCCATGACAACTTTG-3' and 5'-GGGCCATCCACAGTCTTCTG-3'

Immunoblotting:

Cells were lysed in 150 mM NaCl, 50 mM Tris-HCl (pH 7.4), 50mM Glycerophosphate (Sigma-Aldrich, UK), 1% Tween-20, 0.2% Igepal (Sigma-Aldrich, UK) in the presence of protease inhibitor cocktail 2 (Sigma-Aldrich, UK) and phosphatase inhibitor cocktail (Sigma-Aldrich, UK). 35μg of protein lysate were resuspended in Laemmli buffer, denatured for 5 minutes at 95˚C, separated by SDS-PAGE and transferred to nitrocellulose membrane (Whatman, USA). Nitrocellulose membranes were immunoblotted with the following primary antibodies: mouse anti-GAPDH (Abcam, UK); rabbit anti-NRP1, rabbit anti-VEGFR2 (Cell Signaling Technology, USA); rabbit anti-TOM20, rabbit anti-MFN1, rabbit anti-MFN2 (Proteintech, USA), mouse monoclonal anti-KDEL (BD, UK), mouse anti-EEA1 (BD, UK), rabbit anti-ABCB8 (Sigma Aldrich, UK), mouse anti-Transferrin-1 (HTF-14) (Novus Biologicals, UK), goat anti-CDH5, rabbit anti-human p16 (C-20) and rabbit anti-mouse p16 (M-156) (Santa Cruz Biotechnology, USA); rabbit anti-SIRT1, rabbit anti-cyclin D1, rabbit anti-cyclin D2, (Cell Signaling Technology, USA). HRP-conjugated secondary antibodies were used for chemiluminescence detection with ECL prime (Amersham, UK) and protein levels were quantified by densitometry with ImageJ (NIH, Bethesda US).

Immunoprecipitation:

ECs were lysed in 50 mM Tris, pH 8.0, 50 mM KCl, 1% (vol/vol) NP-40 in the presence of protease inhibitor cocktail 2 and phosphatase inhibitor cocktail. 1mg of protein was incubated with 3μg goat anti-NRP1 (Raimondi et al., 2014), or control goat or rabbit IgG (Santa Cruz Biotechnology) and then with 30 μl protein G Sepharose 4 fast flow (GE Healthcare, UK) at 4°C. Beads were washed three times with lysis buffer on a rotating wheel at 4°C for 5 minutes and resuspended in 50μl Laemmli sample buffer for SDS-PAGE and immunoblotting.

Mitochondrial Fractionation:

Mitochondrial fractionation was performed using the Qproteome Mitochondria Isolation Kit (Qiagen, UK). Briefly, 12 million of HDMECs were suspended in Lysis Buffer, centrifuged and the resulting pellet was resuspended in Disruption Buffer. The pellet was disrupted by repeatedly passing through a 23G blunt ended needle to ensure complete cell disruption and recentrifuged to pellet nuclei, cell debris, and unbroken cells. Then, the supernatant containing mitochondria and the microsomal fraction was centrifuged to pellet mitochondria. Mitochondria were washed and resuspended in Mitochondria Storage Buffer and carefully pipetted on top of layers of a gradient purification buffer which allowed the mitochondria to form a band. The band was removed, and the high-purity mitochondria lysed and analysed by immunoblotting for NRP1, GAPDH, ABCB8, TOM20, KDEL and EEA1. Mouse monoclonal [10C3] anti-KDEL (BD, UK) and mouse monoclonal clone 14 anti-EEA1 (BD, UK) were used for immunoblotting analysis.

Immunofluorescence:

For experiments with fixed samples, HDMECs were fixed in 4% formaldehyde in PBS for 10 minutes, permeabilised in 0.25% Triton X-100 in PBS for 3 minutes, blocked for 30 minutes in 0.1%BSA in PBS and incubated overnight with the following primary antibodies: Rabbit anti-TOM20, (Proteintech, UK), rabbit anti-ABCB8 (Sigma, UK), mouse anti-NRP1 (R&D Systems, UK), mouse anti-Transferrin-1 (Santa Cruz Biotechnology, US). The following conjugated secondary antibodies were used: Alexa-488 conjugated goat anti-rabbit/anti-mouse or Alexa-594 anti-rabbit/anti-mouse secondary antibodies (ThermoFisher Scientific, UK) and DAPI (Sigma-Aldrich, UK). Images were acquired with a plan apochromat 63X 1.4 NA oil objective on an LSM780 confocal microscope (Zeiss, Germany). Confocal z-stacks were acquired with an LSM780 laser scanning confocal microscopes (Zeiss, Germany). Maximal intensity projections were generated with ImageJ (NIH, Bethesda, USA) and normalized to DAPI pixel intensity. Mitochondrial areas and volumes per cell were calculated by generating a 3D-rendered surface based on Mitotracker staining with IMARIS (Bitplane, AG). For colocalization analysis, z-stacks acquired with an LSM780 laser scanning confocal microscopes (Zeiss) were deconvoluted with Huygens Deconvolution software (Scientific Volume Imaging B.V., The Netherlands)

and analysed for colocalization using the intensity correlation analysis plug-in for ImageJ (NIH, Bethesda, USA); 3D projection was generated and analysed with Volocity (PerkinElmer, UK).

Whole-mount aorta:

Aortas were obtained by dissection from *Nrp1*^{WT} or *Nrp1*^{ECKO} mice, fixed in 4% paraformaldehyde for 5 minutes, permeabilised with 1:1 Methanol:Acetone for 5 minutes and blocked with 3% BSA in PBS-T for 1 hour. Samples were incubated overnight in 3% BSA in PBS-T with goat anti-NRP1 (R&D, UK) and rabbit anti-PECAM (Invitrogen, UK) or with goat anti-ABCB8 (Santa Cruz Biotechnology, US) and rabbit anti-PECAM. Primary antibodies were detected with Alexa-488 or Alexa-555 anti-rabbit/anti-goat secondary (ThermoFisher Scientific, UK). Nuclei were counterstained with DAPI. Z-projection were generated with ImageJ (NIH, Bethesda, USA) and z-stack pixel intensity was analysed with ImageJ (NIH, Bethesda, USA).

Mitosox live-staining:

ECs were incubated with 5µM Mitosox (Sigma-Aldrich, UK) and 5µg/ml Hoescht 33342 (Sigma-Aldrich, UK) in HBSS with Ca^{2+} and Mg^{2+} (Life Technology, UK) for 8 minutes, washed three times with HBSS with Ca^{2+} and Mg^{2+} before imaging with an LSM780 confocal microscope with a plan apochromat 63X 1.4 NA oil objective (Zeiss, Germany). In some cases, cells were treated with 100µM Deferoxamine (Sigma-Aldrich, UK) for 24 hours before imaging. Mitosox and Hoescht 33342 integrated density was determined with ImageJ (NIH, Bethesda, USA).

CellROX:

Zebrafish embryos were raised at 28.5^{0}C in fish water and to prevent pigment formation, 0,003% phenylthiourea (PTU, Sigma) was added to the fish water at 10hpf. 3dpf embryos were incubated in the dark with 2.5 µM CellROX (Invitrogen) in 10% DMSO for 45 minutes. Followed CellROX staining, embryos were anaesthetised with MS-222 (Sigma-Aldrich, UK), immobilised in 1.2% low melting point agarose (Sigma-Aldrich, UK) and imaged from a lateral perspective using a Leica SP8 confocal microscope. During confocal analysis, we excluded territories containing pigment cells as, even after PTU treatment, showed autofluorescence that could not be

distinguished from CellROX labelling. CellROX staining in the vasculature was quantified with IMARIS (Bitplane, AG) by applying a virtual mask to the z-stacks acquired by high-resolution confocal microscopy to isolate GFP-positive vascular territories. Thus, cellROX signal within the vasculature was quantified and normalized to GFP signal.

Tetramethylrhodamine methyl ester (TMRM) live-staining:

ECs were incubated with 100nM TMRM for 30 minutes in growth MV2 media (HDMECs) or EGM2 (HUVECs), washed three times with HBSS with Ca^{2+} and Mg^{2+} before imaging with an LSM 510 confocal microscope with a plan apochromat 63X 1.4 NA oil objective (Zeiss, Germany). In some cases, cells were nuclear counterstained using DRAQ5 (ThermoFisher, UK) or treated with 100µM Deferoxamine (Sigma-Aldrich, UK) for 24 hours before imaging. TMRM integrated density was determined with ImageJ (NIH, Bethesda, USA) and normalized to the number of cells. In some experiments, ECs were incubated with 100nM TMRM and 300nM Mitotracker Deep Red to simultaneously visualise mitochondria and mitochondrial membrane potential and live-imaged with an LSM 510 confocal microscope with a plan apochromat 63X 1.4 NA oil objective (Zeiss, Germany). TMRM and Mitotracker integrated density was determined with ImageJ (NIH, Bethesda, USA) and expressed as TMRM/Mitotracker ratio.

Mitotracker staining:

ECs were incubated with serum-free MV2 media (Promocell, Germany) containing 500nM MitoTracker™ Orange (ThermoFisher, UK) for 30 minutes. Cells were washed three times in HBSS Ca^{2+} and Mg^{2+} (Life Technology, UK), fixed in 4% PFA for 10 minutes at RT, nuclear counterstained with DAPI and imaged with an LSM710 confocal microscope with a plan apochromat 63X 1.4 NA oil objective (Zeiss, Germany).

Mito-FerroGreen live-staining:

ECs were washed three times with HBSS supplemented with Ca^{2+} and Mg^{2+} and incubated for 30 minutes with 5µM Mito-FerroGreen and 300nM Mitotracker Deep Red. Then, ECs were washed three times with HBSS supplemented with Ca^{2+} and Mg^{2+} and live-imaged with a widefield microscope with a plan apochromat 40X air

objective (Zeiss, Germany) or with an LSM780 confocal microscope with a plan apochromat 63X 1.4 NA oil objective (Zeiss, Germany). Mito-FerroGreen pixels were determined with ImageJ (NIH, Bethesda, USA) and normalized to the number of cells.

Intracellular Iron assay:

Intracellular iron of HDMEC transfected with si-control or si-NRP1 for 72 hours was measured using the colourimetric Iron Assay Kit (Catalog #K390-100 Biovision, US) following the manufacturer's instructions. The kit measures Ferrous and Ferric ion in tissues or cell samples (Bai et al., 2017). In the assay, ferric carrier proteins will dissociate ferric iron into solution in the presence of acid buffer. After reduction to the ferrous form (Fe^{2+}), iron reacts with Ferene-S to produce a stable coloured complex with absorbance at 593 nm.

Proximity ligation assay:

HDMECs were grown on gelatinised coverslips in 8-well chambers (Labtek, Sigma-Aldrich, UK). Cells were fixed with 4% paraformaldehyde for 15 minutes prior to blocking with 3% BSA for 1 h. PLA was performed following the manufacturer's instructions (Duolink, Sigma-Aldrich, UK) using primary mouse anti-NRP1 (antibody R&D Systems, UK), goat anti-ABCB8 (Santa Cruz Biotechnology, USA), rabbit anti-TOM20 (Proteintech, UK) and mouse anti-phosphoserine antibody (clone PSR-45, P5747, Sigma-Aldrich, UK). As control isotype mouse IgG (Santa Cruz Biotechnology, USA) and goat IgG (Santa Cruz Biotechnology, USA) were respectively used in combination with goat anti-ABCB8 or mouse anti-NRP1 or rabbit anti-TOM20. Cells were mounted with Vectashield (Vector Laboratories, USA) and imaged with an LSM780 confocal microscope with a plan apochromat 63X 1.4 NA oil objective (Zeiss, Germany). PLA signal was analysed with the Analyze Particle function of ImageJ (NIH, Bethesda, USA).

BrdU and apoptosis analysis by FACS:

HDMECs were incubated with 10μM BrdU (Sigma) for 30 minutes, washed with PBS and harvested 48 hours after BrdU incorporation. Cells were then washed in PBS, trypsinized, pelleted and resuspended in ice-cold 70% ethanol for 30 minutes at 4°C. Fixed samples were then pelleted, resuspended in 2N HCl at room temperature for 30 minutes. Cells were washed in PBS supplemented with 0.1% BSA

and 0.2% Tween-20, pH 7.4, incubated with 2µl anti-BrdU (Becton Dickinson) at room temperature for 20 minutes, then washed in PBS-Tween and incubated with secondary antibody conjugated with Alexa (Molecular Probes) for 20 minutes at room temperature. Samples were washed in PBS, incubated with 50 µl RNAse (100 µg/ml, Sigma) at 37°C for 15 minutes and then incubated with Propidium iodide (50 µg/ml, Sigma). Apoptosis was assessed by flow cytometry by using annexin V and propidium iodide staining. Cells were washed in PBS, trypsinized, pelleted and resuspended in 400 µl of binding buffer (Becton Dickinson, San Jose, CA); samples were incubated with 2 µl of annexin-V–FITC (Becton Dickinson) for 15 minutes at room temperature, then propidium iodide (5 µg/ml) was added to the sample.

SA-β-gal assay:

SA-β-gal activity was identified with the Senescence Cells Histochemical Staining Kit (catalogue number CS0030; Sigma-Aldrich, UK). Briefly, ECs were fixed for 6 minutes in 1X fixation buffer (2% formaldehyde, 0.2% glutaraldehyde, 7.0 mM Na2HPO4, 1.5 mM KH2PO4, 0.13 M NaCl, and 2.68 mM KCl), washed twice with PBS and then incubated for 16 hours at 37°C with SA-β-gal staining solution comprised of 5-bromo-4-chloro-3-indoyl β-D-galactopyranoside (X-Gal; 1mg/ml, diluted from a 40 mg/ml stock solution in N,N-dimethylformamide), potassium ferrocyanide (5 mM) and potassium ferricyanide (5 mM) in PBS (pH 6.0.) Following incubation, cells were washed three times with PBS and viewed under phase contrast using a Nikon Eclipse TS100 microscope with a 10x objective (N.A. 0.3, W.D. 16.0 mm). Senescent ECs were identified by the presence of obvious blue reaction product. For each sample, the average percentage of SA-β-gal positive cells in each of three fields of view (0.785 mm2) was calculated as the value for that sample.

PTMScan:

Samples were analyzed using the PTMScan method as previously described (Stokes et al., 2012). Cellular extracts were prepared in urea lysis buffer, sonicated, centrifuged, reduced with DTT, and alkylated9 with iodoacetamide. Equal amounts of total protein for each sample was digested with trypsin and purified over C18 columns for enrichment with the Phosphotyrosine pY-1000 antibody (#8954) or Phospho-Enrichment IMAC Fe-NTA Magnetic Beads (#20432). Enriched peptides were purified over C18 STAGE tips. Replicate injections of each sample were run non-

sequentially on the instrument. Peptides were eluted using a 90- or 150-minute linear gradient of acetonitrile in 0.125% formic acid delivered at 280 nL/min. Tandem mass spectra were collected in a data-dependent manner with an Orbitrap Velos or Elite Hybrid Ion Trap-Orbitrap Mass Spectrometer running XCalibur 2.0.7 SP1 using a top-twenty MS/MS method, a dynamic repeat count of one, and a repeat duration of 30 seconds. Real-time recalibration of mass error was performed using lock mass with a singly charged polysiloxane ion m/z = 371.101237. MS/MS spectra were evaluated using SEQUEST and the GFY-Core platform. Files were searched against the NCBI homo sapiens FASTA database. A mass accuracy of +/-50 ppm was used for precursor ions and 1.0 Da for product ions. Enzyme specificity was limited to trypsin, with at least one tryptic (K- or R-containing) terminus required per peptide and up to four mis-cleavages allowed. Cysteine carboxamidomethylation was specified as a static modification, oxidation of methionine and phosphorylation on serine, threonine, or tyrosine residues were allowed as variable modifications. Reverse decoy databases were included for all searches to estimate false discovery rates and filtered using a 5% FDR in Core. Peptides were also manually filtered using a -/+ 5ppm mass error range and presence of at least one phosphorylated residue on each peptide. All quantitative results were generated using Progenesis V4.1 (Waters Corporation) to extract the integrated peak area of the corresponding peptide assignments. The accuracy of quantitative data was ensured by manual review in Progenesis or in the ion chromatogram files. A 2.5-fold cutoff was used to denote changes between samples and analytical % CV values were calculated for each peptide to determine reproducibility across runs. A limited version of the raw dataset containing only PTMscan data of mitochondria-associated proteins has been made available to readers due to intellectual property concerns.

Statistical analyses:

Statistical analyses were performed with GraphPad (Prism) or Office Excel (Microsoft); data obtained from cell lines were analysed with a two-tailed, paired t-test; data obtained from the zebrafish experiments were analysed with a one-way ANOVA statistical test; data obtained from mouse experiments were analysed with a two-way ANOVA statistical test.

Supplemental Information Figures:

Figure S1 related to Figure 1 and Figure 3

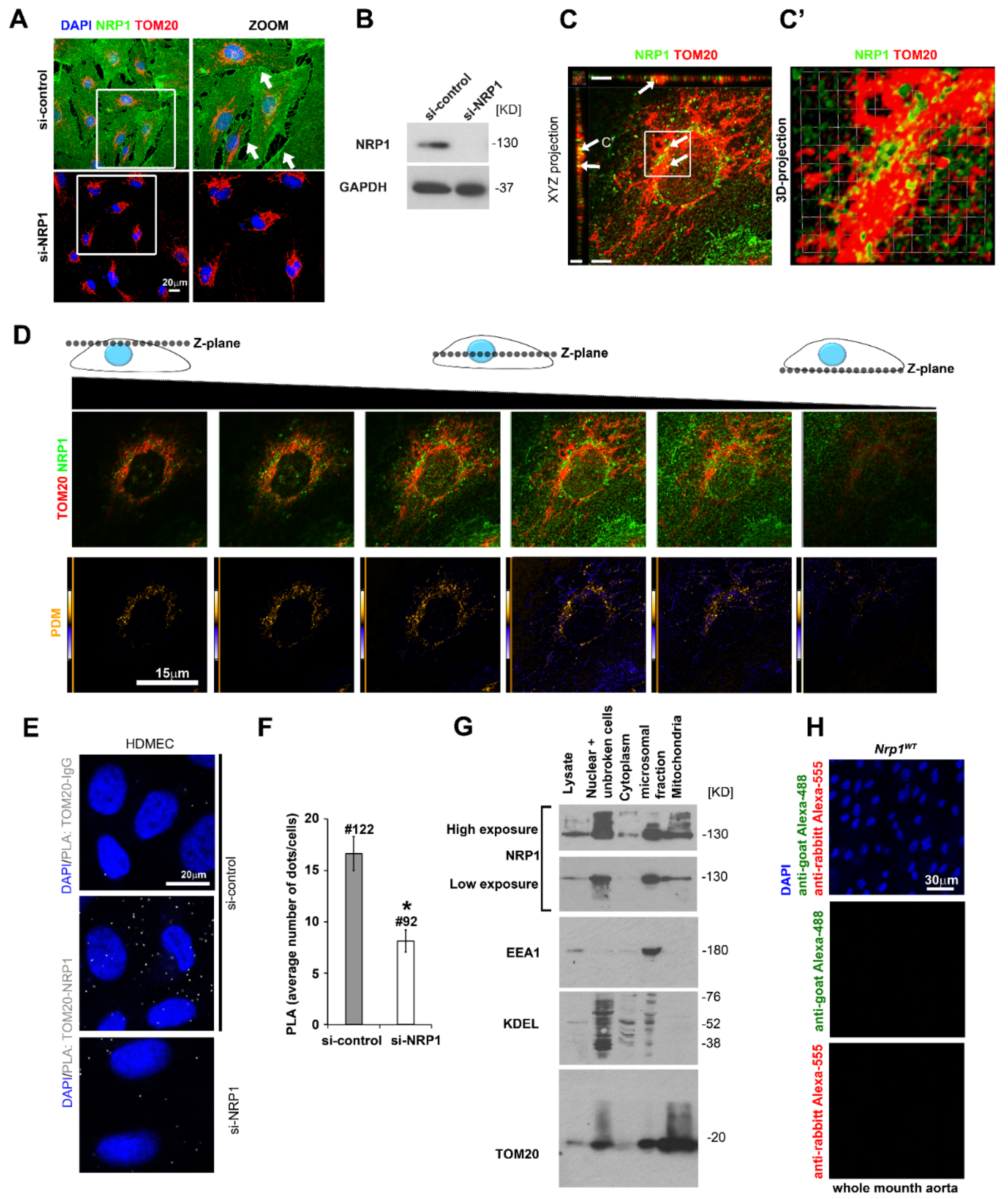

Figure S1 – Related to Figure 1 and Figure 3: A) HDMECs si-control or si-NRP1 immunostained for NRP1, TOM20 and counterstained with DAPI (n=3). Arrows indicate NRP1 staining at the plasma membrane and in filopodia; Scale bars: 20µm. B) Representative immunoblot for NRP1 of HDMECs si-NRP1 or si-control 72 hours with GAPDH used as loading control; (n≥5). C) High magnification orthogonal view of NRP1 and TOM20 double-stained HDMEC showing colocalization (arrows) of NRP1 and TOM20 along X and Y axes; scale bars 3µm and 5µm. C') High magnification of the area indicated in panel C, showing colocalization of TOM20 and NRP1 within three-dimensional structures. D) Colocalization analysis of single z-planes extracted from the z-stack shown in panel C and in Figure 1A; Scale bar 15µm. E) PLA for TOM20 and NRP1 or TOM20 and IgG isotype of HDMECs si-control and si-NRP1 analysed using a confocal microscope; Scale bars: 20µm. F) PLA signal (grey) per cells (mean ± SEM) was measured in a minimum of 92 cells from 2 independent experiments. G) Fractions isolated with Qproteome mitochondria isolation kit from HDMECs were immunoblotted for the indicated antibodies (n=3). H) Aortas of *Nrp1*$^{fl/fl}$ (*Nrp1*^{WT}) injected daily with tamoxifen for 5 days and sacrificed after 1 month from injections stained with secondary only as negative control; bar 30µm. *, $P < 0.05$, Student's t-test.

Figure S2, related to Figure 2 and Figure 3

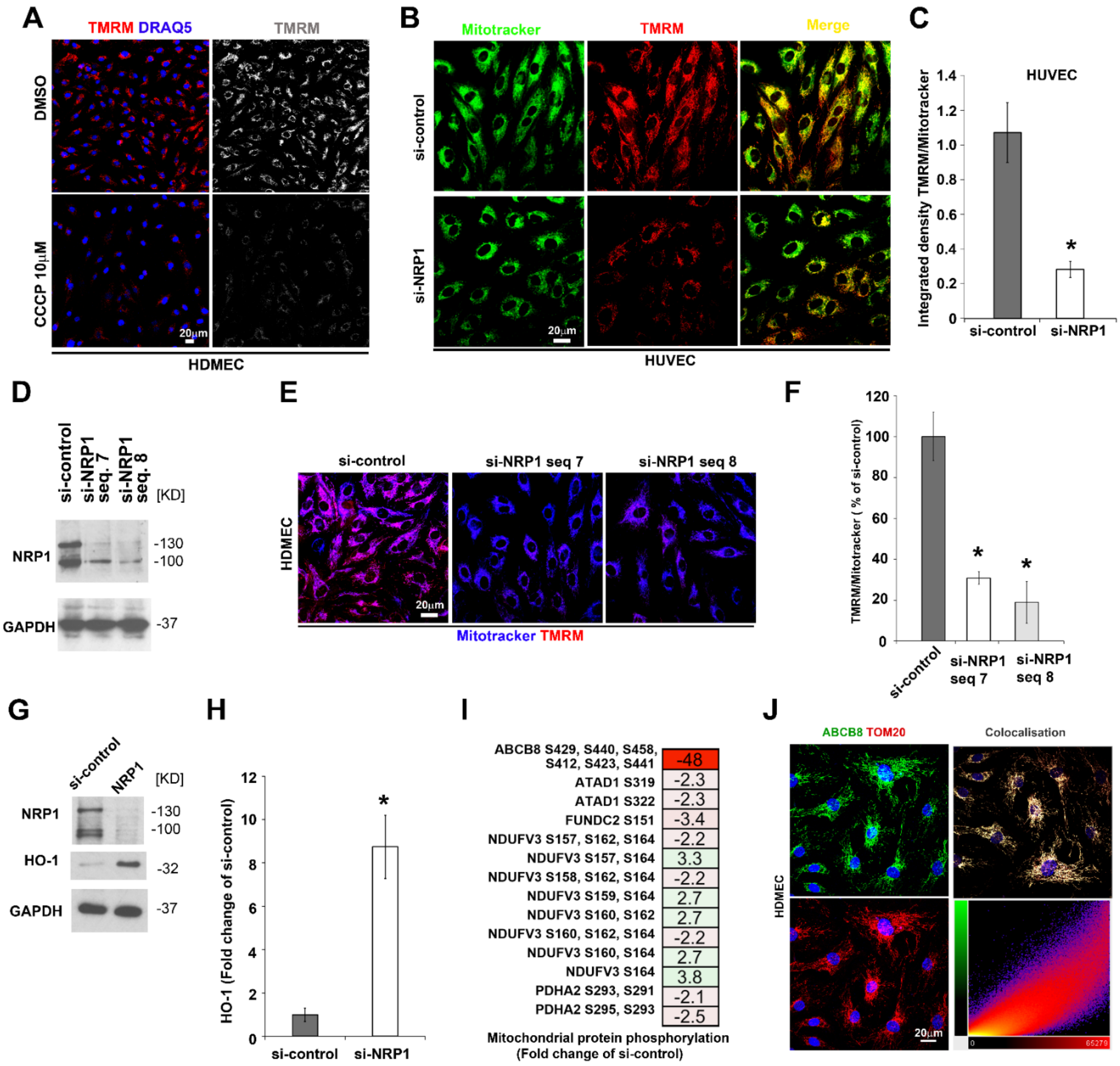

Figure S2 – Related to Figure 2 and Figure 3: A) HDMECs incubated with TMRM 100nM and counterstained with DRAQ5 (blue) were treated or left untreated with 10μM CCCP; n=2; Scale bars: 20μm. B) HUVECs siNRP1 or si-control were incubated with 100nM TMRM and 300nM Mitotracker Deep Red FM and live-imaged with a confocal microscope. C) TMRM and Mitotracker Deep Red FM integrated density was calculated, and the ratio visualised in the graph (mean ± SEM; n=4). D) Representative immunoblotting for NRP1 in lysates from HDMECs transfected with si-control, anti-NRP1 single sequence 7 or single sequence 8 siRNAs. GAPDH was used as loading control (n=3). E) HDMECs transfected with siNRP1 single sequence 7 or single sequence 8 or si-control were incubated with 100nM TMRM and 300nM Mitotracker Deep Red FM and live-imaged with a confocal microscope. F) TMRM and Mitotracker Deep Red FM integrated density was calculated, and the ratio visualised in the graph (mean ± SEM; n=3). G) Representative immunoblots for NRP1, HO-1 and GAPDH in HDMECs si-NRP1 or si-control. H) Quantification of expression levels as pixel intensity relative to GAPDH pixel intensity; n=3. I) Heat map showing data from PTMscan of phosphorylated mitochondrial proteins in HDMECs transfected with si-control or si-NRP1 for 72 hours. Data are expressed as fold change of si-control; n=2. J) HDMECs were co-stained for ABCB8 (green) and TOM20 (red) with DAPI (blue) used as counterstaining. Right panels show a bi-parametric correlative histogram; Scale bars: 20μm. *, $P < 0.05$, Student's t-test.

Figure S3, related to figure 4 and Figure 5

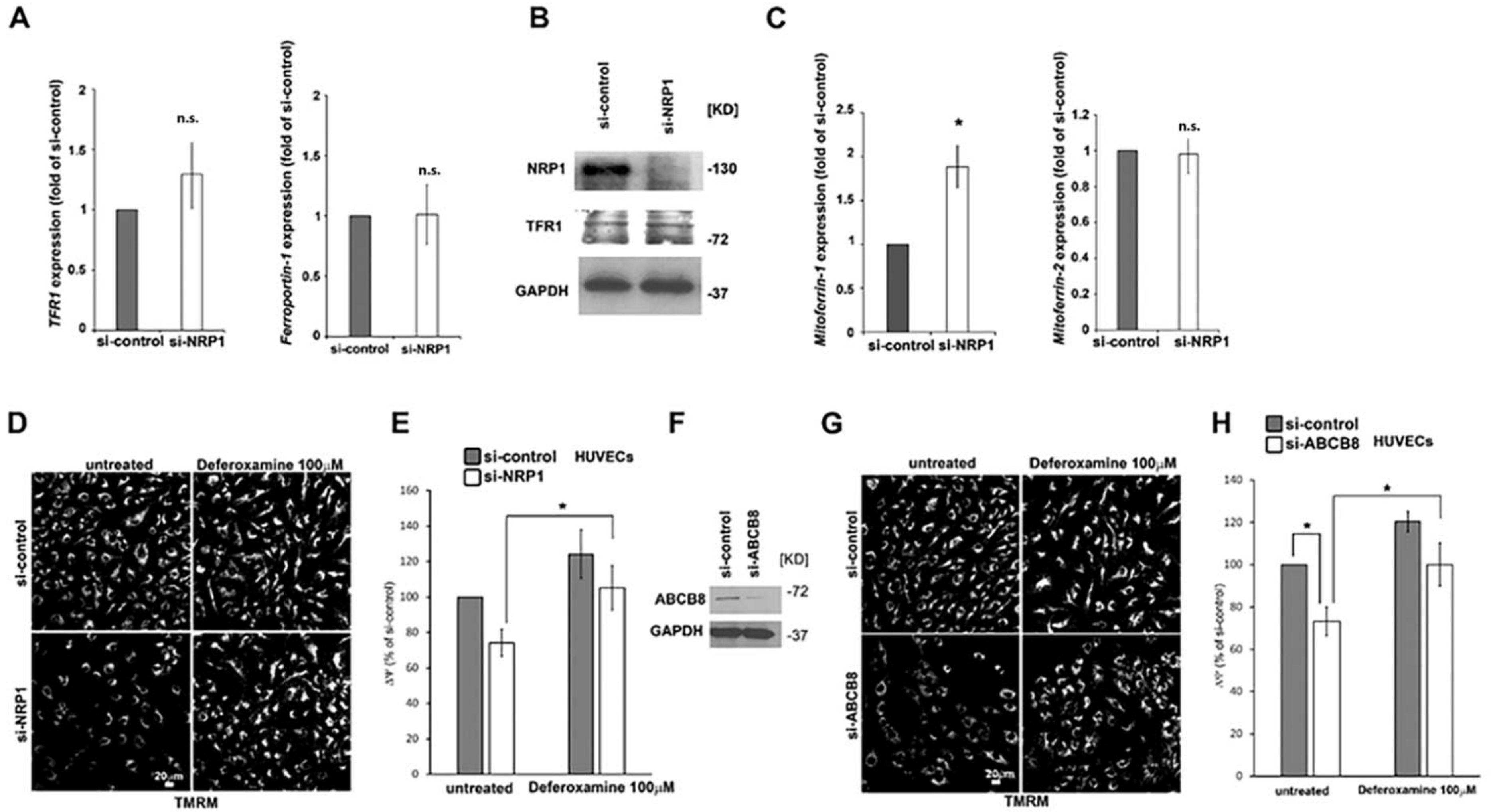

Figure S3 – Related to Figure 4 and Figure 5: A) Quantification of Transferrin-1 (*TFR1*) and *Ferroportin-1* mRNA by RT-qPCR in HDMECs transfected for 72 hours with si-NRP1 or si-control. Transcripts levels were expressed as fold change of si-control (mean ± SEM; n=3). B) Representative immunoblot for NRP1 and Transferrin-1 (TFR1) of lysates of HDMECs si-control or siNRP1. GAPDH was used as equal loading control; n=3. C) Quantification of Mitoferrin-1 and Mitoferrin-2 mRNA by RT-qPCR in HDMECs transfected for 72 hours with si-NRP1 or si-control. Transcripts levels were expressed as fold change of si-control (mean ± SEM; n=3). D) HUVECs treated with Deferoxamine 100μM for 24 hours after 48 hours from transfection with si-NRP1 or si-control were incubated with 100nM TMRM (grey) and live-imaged with a confocal microscope; Scale bars: 20μm. E) Integrated density of the TMRM signal was quantified, normalized to cell number, and expressed as percentage relative to si-control (mean ± SEM; n=3). F) Representative immunoblotting for ABCB8 in HDMECs si-ABCB8 or si-control (n=2). G) HUVECs treated with Deferoxamine 100μM for 24 hours after 48 hours from transfection with si-ABCB8 or si-control were incubated with 100nM TMRM (grey) and live-imaged with a confocal microscope; Scale bars: 20μm. H) Integrated density of the TMRM signal was quantified, normalized to cell number and expressed as percentage relative to si-control (mean ± SEM; n=3). *, $P < 0.05$; n.s. = not significant; Student's t-test.
